# Activation of HTR2B Suppresses Osteosarcoma Progression through the STAT1‐NLRP3 Inflammasome Pathway and Promotes OASL1+ Macrophage Production to Enhance Antitumor Immunity

**DOI:** 10.1002/advs.202415276

**Published:** 2025-05-19

**Authors:** Zhen Huang, Jiazhuang Zhu, Jianping Hu, Xingkai Wang, Xiaolong Ma, Enjie Xu, Kunpeng Zhu, Chunlin Zhang

**Affiliations:** ^1^ Department of Orthopaedic Shanghai Tenth People's Hospital School of Medicine Tongji University Shanghai 200092 China

**Keywords:** activation, HTR2B, OASL1+ macrophage, osteosarcoma, STAT1‐NLRP3 inflammasome

## Abstract

Osteosarcoma is a primary malignant bone tumor originating from mesenchymal tissue, and associated with poor prognosis. The 5‐hydroxytryptamine receptor 2B (HTR2B), a receptor for serotonin, is known to play a role in the progression of multiple tumors. This study aims to explore the potential roles of HTR2B in osteosarcoma progression. HTR2B expression is analyzed using the TARGET, GEO databases, and osteosarcoma tissue samples in the hospital. Lentivirus and agonist BW‐723C86 are employed to assess HTR2B overexpression effects in osteosarcoma cell lines. Transcriptome sequencing analysis and single‐cell sequencing are performed to identify potential downstream molecules and signaling pathways, and the changes in tumor immune microenvironment. The investigation demonstrates that HTR2B is downregulated in osteosarcoma tissues, and correlates with poorer survival outcomes. Upregulating HTR2B through lentiviral‐mediated gene delivery or the agonist BW‐723C86, resulted in a marked suppression of osteosarcoma cell progression via the STAT1‐NLRP3 inflammasome pathway. Single‐cell sequencing of CD45+ cells reveals that HTR2B activation enhances the production of OASL1+ macrophages, contributing to the observed enhancement of antitumor immunity. These findings propose HTR2B as a novel therapeutic target for treating osteosarcoma, offering a dual mechanism of action that directly impedes tumor cell proliferation and augments the host immune response.

## Graphical abstract

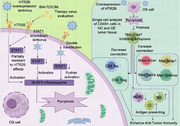

## 1 Introduction

Osteosarcoma is the most common primary bone malignancy, and predominantly affecting adolescents.^[^ ^1^, ^2^, ^3^ ^]^ With advances in chemotherapy and surgical intervention, the overall survival rate for osteosarcoma patients has improved to ≈70%.^[^ ^4^, ^5^, ^6^ ^]^ However, osteosarcoma patients diagnosed with lung metastases or recurrent face a much poorer prognosis, with a 5‐year survival rate of only around 30%.^[^ ^7^ ^]^ Osteosarcoma remains the leading cause of cancer‐related mortality in adolescent, mainly due to its heterogeneity, genomic instability, propensity for early metastasis, and chemotherapy resistance.^[^ ^8^ ^]^ Therefore, unraveling the complex mechanisms underlying osteosarcoma progression is essential for the identification of novel therapeutic targets and the development of more effectively treatment strategies.

The serotonin receptors, a class of G‐protein‐coupled receptors and ligand‐gated ion channels are expressed in various cell types.^[^ ^9^, ^10^ ^]^ These receptors have been implicated in oncogenesis, functioning as potent trophic, mitogenic, and antiapoptotic factors.^[^ ^11^, ^12^ ^]^ Among them, 5‐hydroxytryptamine receptor 2B (HTR2B) is critical in cell viability and proliferation.^[^ ^13^ ^]^ Notably, HTR2B has been shown to exert dual effects depending on the cancer stage, for example, it inhibits the development of colitis‐associated cancers through mediated serotonin activation in intestinal cells at an early stage, but promotes cancer progression at an advanced stage.^[^ ^14^ ^]^ The expression and function of HTR2B vary across tumors types, suggesting its role is context‐dependent.^[^ ^15^ ^]^ To date, the impact of HTR2B on osteosarcoma progression has not been reported.

Current immunotherapeutic approaches for osteosarcoma primarily focuses on immune checkpoint inhibitor, particularly monoclonal antibodies targeting cytotoxic T lymphocyte monoclonal antibody and programmed death‐1/programmed death‐ligand 1 (PD‐1/PD‐L1), but the outcomes have been suboptimal.^[^ ^16^, ^17^ ^]^ Additionally, cytokine‐based immunotherapies, which involve the stimulation of immune cells with cytokines, such as interleukin‐8 (IL‐8) and tumor necrosis factor‐alpha (TNF‐α), are limited by significant toxicities and side effects.^[^ ^18^, ^19^ ^]^ Despite these challenges, the potential of immunotherapy in osteosarcoma remains promising, underscoring the importance of exploring the tumor immune microenvironment in this context. Previous studies suggest that HTR2B may play a role in modulating the tumor microenvironment.^[^ ^20^, ^21^ ^]^ Therefore, this study further investigated the relationship between HTR2B and the immune microenvironment in osteosarcoma.

## 2 Results

### 2.1 The Expression and Prognostic Value of HTR2B in Osteosarcoma

**Figure 1:**
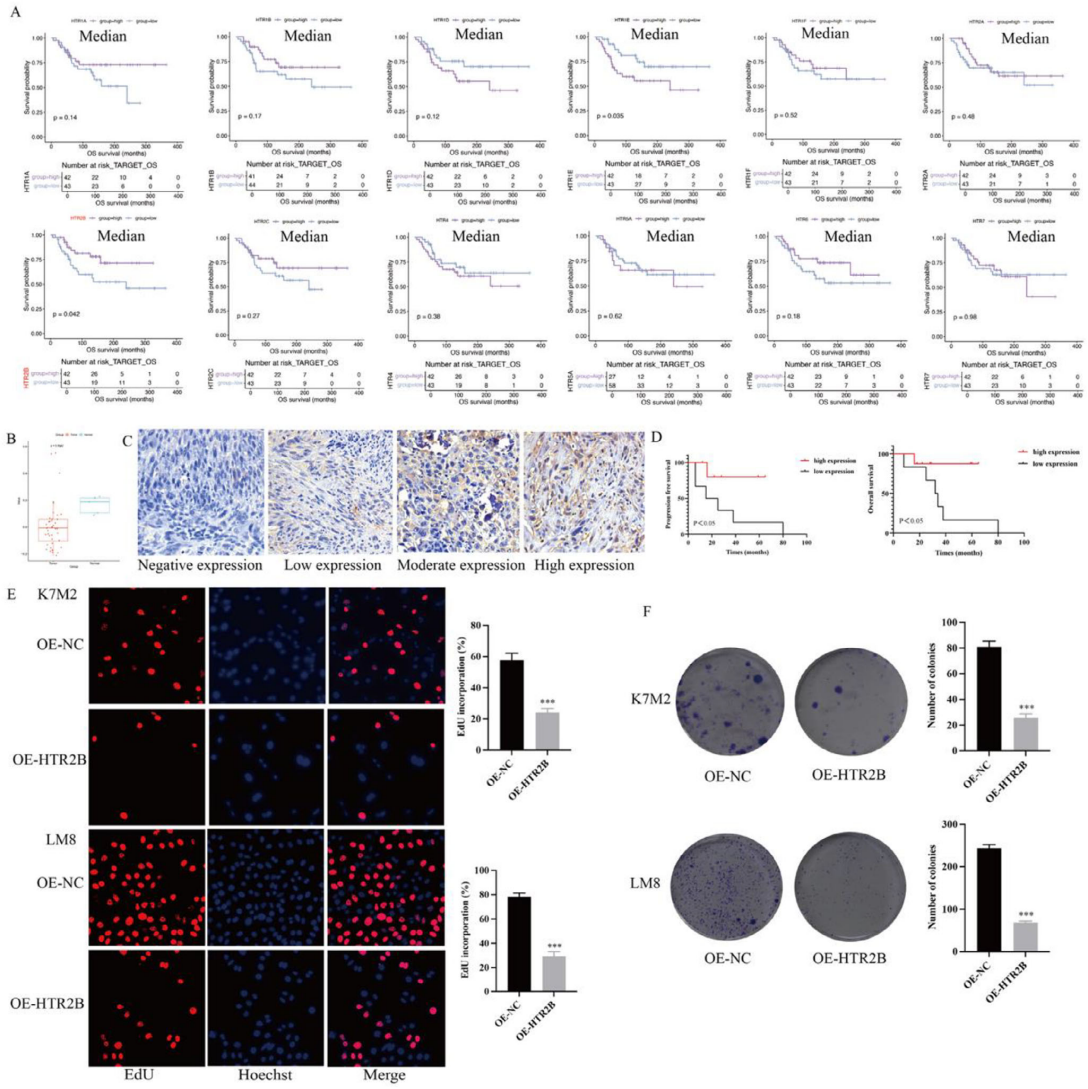
The expression of HTR2B in osteosarcoma and its relationship with the prognosis of patients with osteosarcoma, and the effect of HTR2B overexpression on the proliferation of osteosarcoma cells. A) Analysis of serotonin receptors with osteosarcoma prognosis in TARGET‐Osteosarcoma database revealed that low expression of HTR2B related to poor prognosis. B) Through the public database analysis found that HTR2B expression was downregulated in osteosarcoma. C) Our hospital osteosarcoma samples confirmed low expression of HTR2B in osteosarcoma tissue. D) Our hospital osteosarcoma samples found that low expression of HTR2B closely related to poor prognosis of osteosarcoma patients. E) EdU assay revealed that the proliferation capacity of K7M2 and LM8 cells in HTR2B overexpression group was significantly reduced. F) Colony formation found that HTR2B overexpression suppress the colony formation ability. ***P < 0.001.

Analysis of the association between serotonin receptors (HTR1A, HTR1B, HTR1D, HTR1E, HTR1F, HTR2A, HTR2B, HTR2C, HTR4, HTR5A, HTR6, and HTR7) and osteosarcoma prognosis in the TARGET‐Osteosarcoma database revealed that low expression of HTR2B correlated with patient poor outcomes (**Figure** **1**A). The GSE19276 dataset revealed that HTR2B is downregulated in osteosarcoma tissues compared to normal tissues (Figure 1B). In addition, validation using a human osteosarcoma tissue corroborated these findings, Figure 1C showed the differential expressions of HTR2B protein in osteosarcoma samples. Survival analysis was conducted in osteosarcoma samples, and reinforcing the prognostic significance of low HTR2B expression as an indicator of adverse survival in osteosarcoma patients (Figure 1D).

### 2.2 Overexpression of HTR2B Suppresses Osteosarcoma Cell Proliferation, Migration, and Invasion, Arrests the Cell Cycle In Vitro, and Reduces Tumor Growth In Vivo

**Figure 2:**
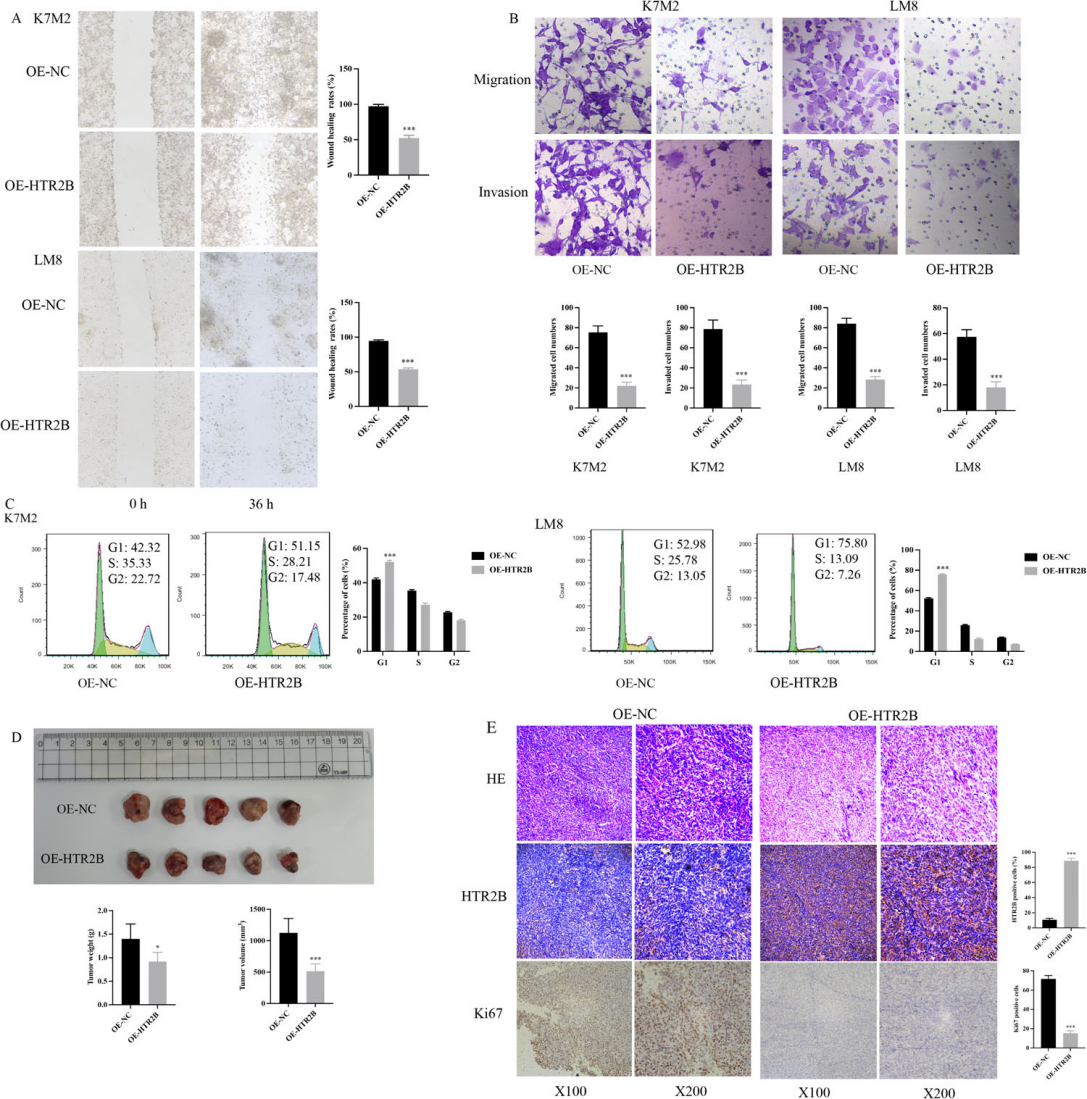
Effects of HTR2B overexpression on osteosarcoma cell migration, invasion, cell cycle, and tumor growth. A) Wound healing showed that HTR2B overexpression group of K7M2 and LM8 cells inhibited cell migration. B) Transwell assay revealed reduced migration and invasion abilities of K7M2 and LM8 cells with HTR2B overexpression. C) Increased proportion of G1 phase in the K7M2 and LM8 cells was observed with HTR2B overexpression. D) Tumor growth in vivo was significantly inhibited in the HTR2B overexpression group. E) The HE staining confirmed that osteosarcoma tumor tissue in OE‐NC and OE‐HTR2B group, and HTR2B expression is promoted in OE‐HTR2B group, Ki67 protein is decreased in OE‐HTR2B group. *P < 0.05, ***P < 0.001.

Lentivirus transfection was employed to overexpress HTR2B in osteosarcoma cells, as shown in Figure S1A (Supporting Information). The quantitative real‐time polymerase chain reaction (RT‐qPCR) and western blot analysis confirmed a significant increase in HTR2B expression in the OE‐HTR2B group compared to the OE‐NC group (Figure S1B,C, Supporting Information). EdU and colony formation assays showed that HTR2B overexpression obviously decreased cell proliferation in K7M2 and LM8 cells (Figure 1E,F). Wound healing assay indicated a reduction in cell migration ability in the OE‐HTR2B group compare to the OE‐NC group (**Figure** **2**A). Transwell assay further revealed that HTR2B overexpression diminished both migration and invasion capabilities of osteosarcoma cells (Figure 2B). Cell cycle detection revealed that HTR2B overexpression can lead to cell cycle arrest in the G1 phase in K7M2 and LM8 cell lines (Figure 2C). In vivo experiments demonstrated that the HTR2B overexpression by lentivirus can suppress tumor growth in vivo (Figure 2D). The HE staining confirmed the presence of osteosarcoma tumor tissues, the immunohistochemical staining revealed increased HTR2B expression, and decreased Ki67 levels in the OE‐HTR2B group (Figure 2E). These findings suggested that activation of HTR2B by lentivirus can suppress tumor proliferation in vivo.

### 2.3 Activation of HTR2B by BW‐723C86 Suppresses Proliferation, Migration, and Invasion, Arrests the Cell Cycle In Vitro, and Reduces Tumor Growth In Vivo

**Figure 3:**
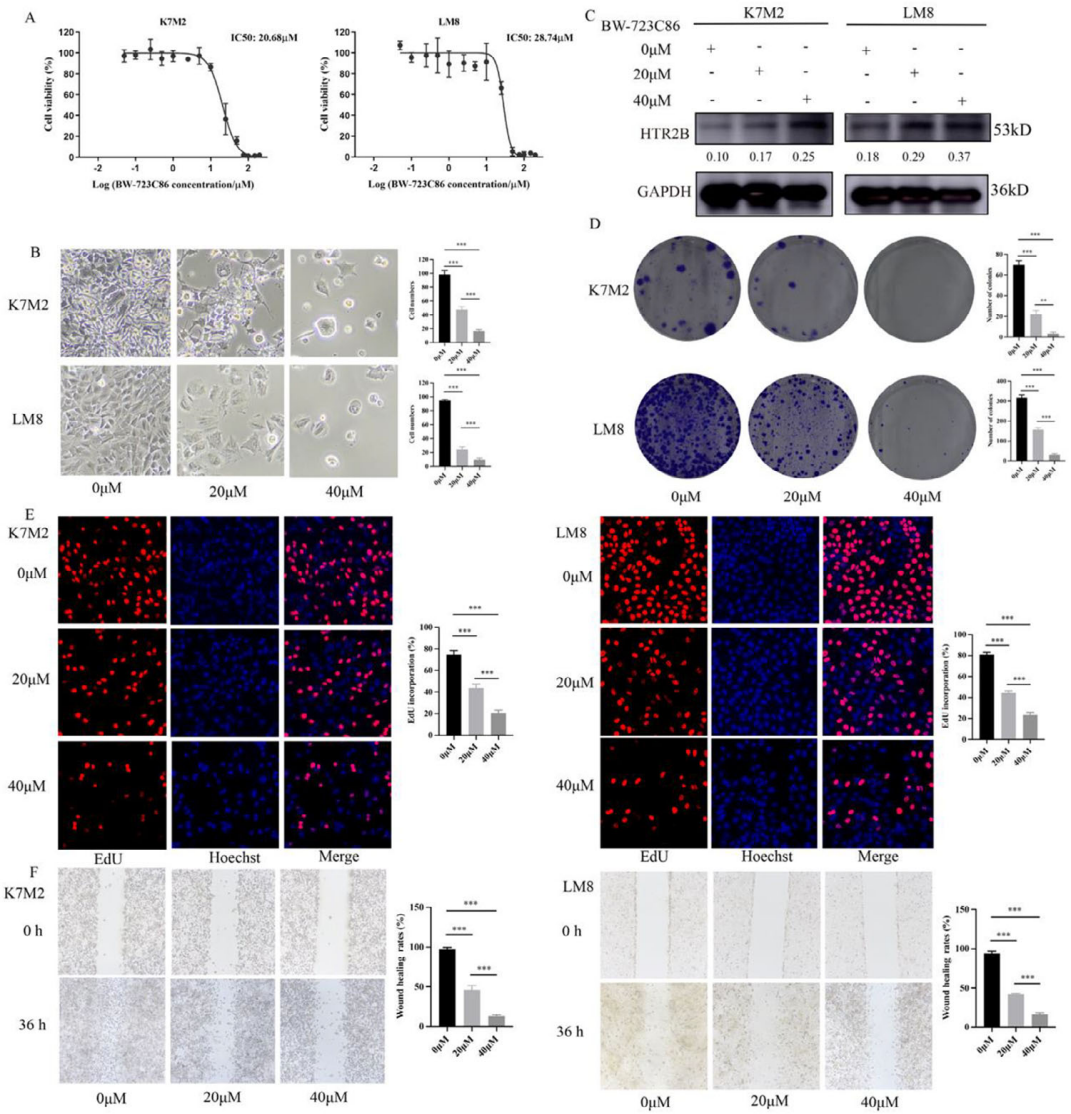
Effects of HTR2B agonist (BW‐723C86) on osteosarcoma cell activity, proliferation, and migration. A) Changes of cell viability of K7M2 and LM8 cells at different concentrations of BW‐723C86. B) Effects of BW‐723C86 on the state of K7M2 cells and LM8 cells after 48 h intervention at 0, 20, and 40 µm concentrations. C) BW‐723C86 treatment increased HTR2B expression in K7M2 and LM8 cells. D) Colony formation ability of K7M2 and LM8 cells was inhibited with increasing BW‐723C86 concentration. E) The proliferation ability of K7M2 and LM8 cells was gradually inhibited with the increase of BW‐723C86 concentration. F) The migration ability of K7M2 and LM8 cells was gradually inhibited with the increase of BW‐723C86 concentration. ***P <0.001.

**Figure 4:**
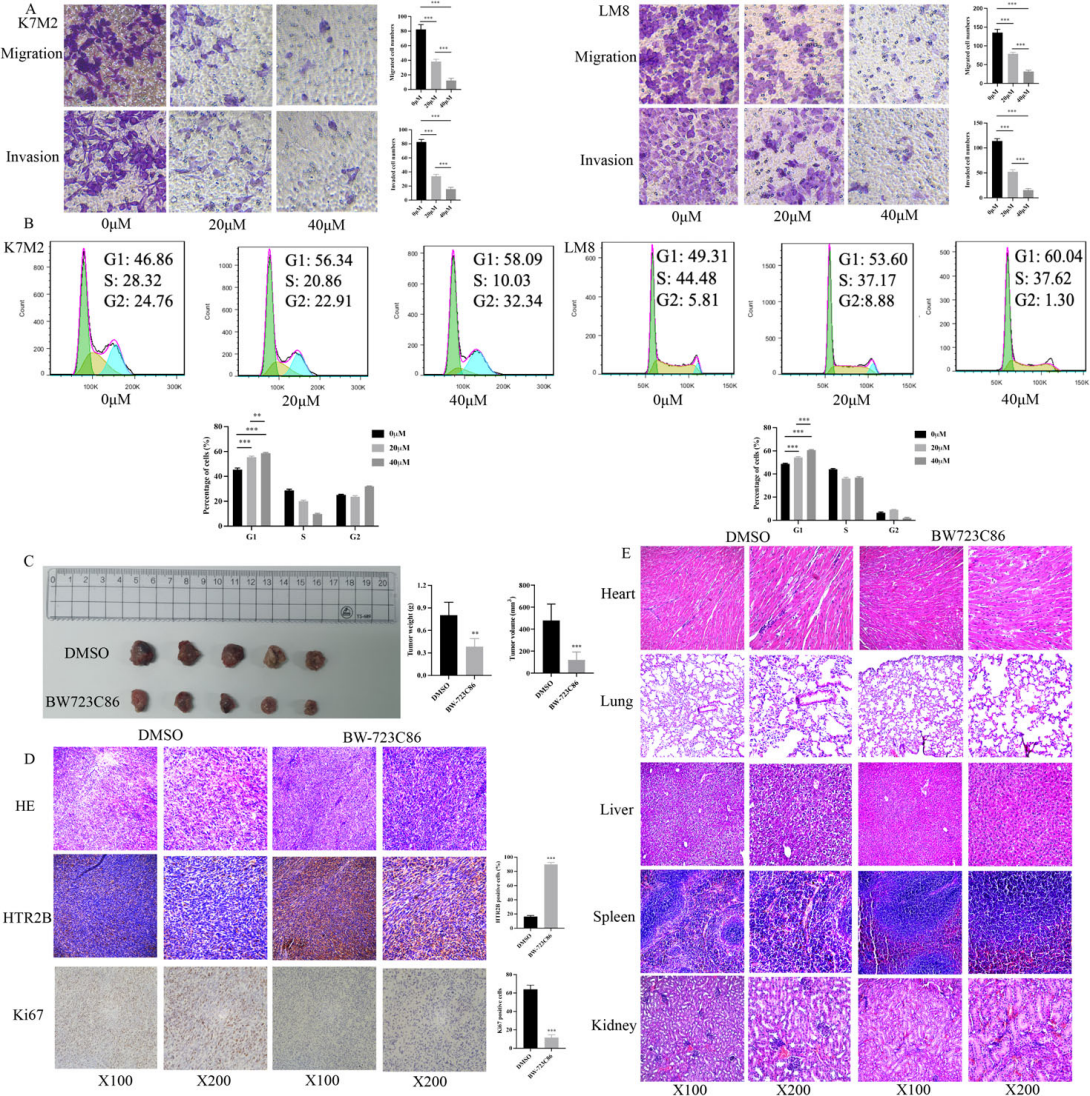
BW‐723C86 effects on cell migration, invasion, cell cycle, and tumor growth in vivo. A) Migration and invasion abilities of K7M2 and LM8 cells were inhibited by BW‐723C86 in a dose‐dependent manner. B) The G1 phase proportions of K7M2 and LM8 cells gradually increased with the increase of BW‐723C86 concentration. C) BW‐723C86 treatment significantly inhibited tumor growth in vivo compared with DMSO control group. D) The HE staining confirmed that osteosarcoma tumor tissue in BW‐723C86 and DMSO group, and HTR2B expression is promoted in BW‐723C86 group, Ki67 protein is decreased in BW‐723C86 group. E) No significant visceral toxicity observed in heart, lung, liver, spleen, and kidney after BW‐723C86 intervention. ***P < 0.001.

To evaluate the therapeutic potential of targeting HTR2B, the selective agonist BW‐723C86 was used. The CCK‐8 assay indicated that BW‐723C86 effectively inhibits the cell viability of K7M2 and LM8 cells (**Figure** **3**A). Based on the dose‐response curves, concentrations of 0, 20, and 40 µm were selected for further investigation. As shown in Figure 3B, increased concentrations of BW‐723C86 resulted in a significant decrease in cell viability. The western blot analysis confirmed that increased concentrations of BW‐723C86 promote the expression of HTR2B protein (Figure 3C). Colony formation and EdU assays revealed that cell proliferation was inhibited with the increased concentrations of BW‐723C86 (Figure 3D,E). Wound healing assay demonstrated that BW‐723C86 effectively reduced cell migration (Figure 3F). Transwell assay further confirmed that BW‐723C86 treatment decreased both migration and invasion abilities in osteosarcoma cells (**Figure** **4**A). Moreover, the flow cytometry experiment showed that BW‐723C86 induced cell cycle arrest at the G1 phase (Figure 4B). The in vivo experiment demonstrated that BW‐723C86 significantly reduced tumor growth, and prolong survival rates (Figure 4C; and Figure S2, Supporting Information). HE staining confirmed the presence of osteosarcoma tumor tissues, IHC revealed increased HTR2B expression and reduced Ki67 levels in the BW‐723C86‐treated group (Figure 4D). Additionally, HE staining of heart, lung, kidney, spleen, and liver tissues showed no significant toxic effects of BW‐723C86 on these organs (Figure 4E).

### 2.4 Inhibition of HTR2B by RS‐127445 Promotes Proliferation, Migration, and Invasion, Accelerates the Cell Cycle In Vitro, and Increases Tumor Growth In Vivo

HTR2B inhibitor RS‐127445 was used to verify the specific action of HTR2B and exclude nonspecific effects. The RT‐qPCR analysis confirmed that RS‐127445 treatment significantly decreased the expression of HTR2B (Figure S3A, Supporting Information). Colony formation and EdU assays revealed that cell proliferation was promoted by the treatment of RS‐127445 (Figure S3B,C, Supporting Information). Wound healing and transwell assay further confirmed that RS‐127445 treatment increased migration and invasion abilities in osteosarcoma cells (Figure S3D,E, Supporting Information). Moreover, the flow cytometry experiment indicated that RS‐127445 treatment decreased at the G1 phase in the cell cycle (Figure S3F, Supporting Information). The in vivo experiment demonstrated that RS‐127445 treatment promoted tumor growth (Figure S3G, Supporting Information).

### 2.5 Activation of HTR2B Suppresses Osteosarcoma Progression may be Reversed by STAT1

**Figure 5:**
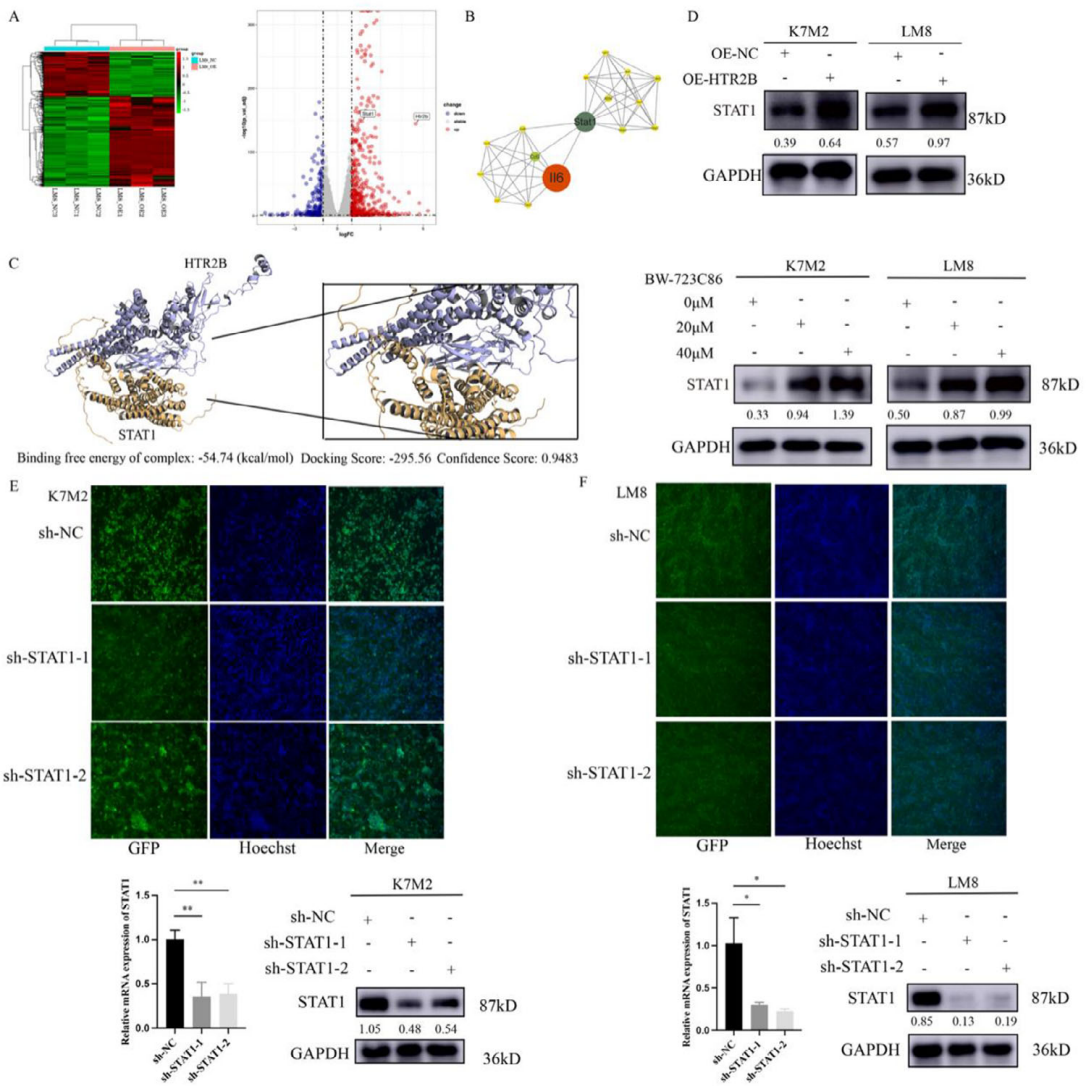
Activation of HTR2B promotes STAT1 protein expression in osteosarcoma cell lines. A) Transcriptome sequencing analysis showed that HTR2B and STAT1 genes were significantly upregulated in HTR2B overexpression group. B) The protein–protein interaction analysis suggested STAT1 may be potential downstream target of HTR2B activation. (C) HDOCK Server analysis showed that HTR2B and STAT1 can be stable binding. D) Western blot analysis demonstrated that HTR2B activation by lentivirus or BW‐723C86 increased STAT1 protein expression. E) Representative images of STAT1 lentivirus transfection in K7M2 cell and mRNA and protein expression of STAT1 were significantly decreased. F) The mRNA and protein expression of STAT1 were significantly decreased in STAT1 lentivirus transfection in LM8 cell. *P < 0.05, **P < 0.01.

The RNA sequence analysis in LM8‐OE‐NC (NC) and LM8‐OE‐HTR2B (OE) cells identified upregulated genes (containing HTR2B and STAT1) and down‐regulated genes in the OE‐HTR2B group compared to the OE‐NC group (**Figure** **5**A). Protein–protein interaction analysis suggested that STAT1 may be a potential downstream‐acting molecule of HTR2B activation suppresses osteosarcoma progression (Figure 5B). The GSEA enrichment analysis found that the cellular response to the interferon‐gamma pathway is enriched, and the gene expression analysis revealed that multiple interferon‐related molecules are activated (Figure S4, Supporting Information). HDOCK Server was used to predict the near‐natural structure of the complex by molecular simulation based on the 3D structure of HTR2B and STAT1 proteins. The results showed that the binding free energy of the complex is −54.74 (kcal mol^−1^) with a docking score −295.56 and a confidence score of 0.9483 which suggested that HTR2B and STAT1 can be stable binding (Figure 5C). The western blot analysis confirmed that STAT1 protein is significantly promoted in the OE‐HTR2B and BW‐723C86 treatment groups of K7M2 and LM8 cells (Figure 5D). To further investigate the role of STAT1 in osteosarcoma, we used the lentivirus to downregulate the expression of STAT1, the RT‐qPCR and western blot results confirmed that the mRNA and protein expression STAT1 is effectively downregulated after lentivirus transfection in osteosarcoma cells (Figure 5E,F).

### 2.6 Downregulated of STAT1 Suppresses Proliferation, Migration, Invasion, and Arrests the Cell Cycle In Vitro, and Reduce Tumor Growth In Vivo

**Figure 6:**
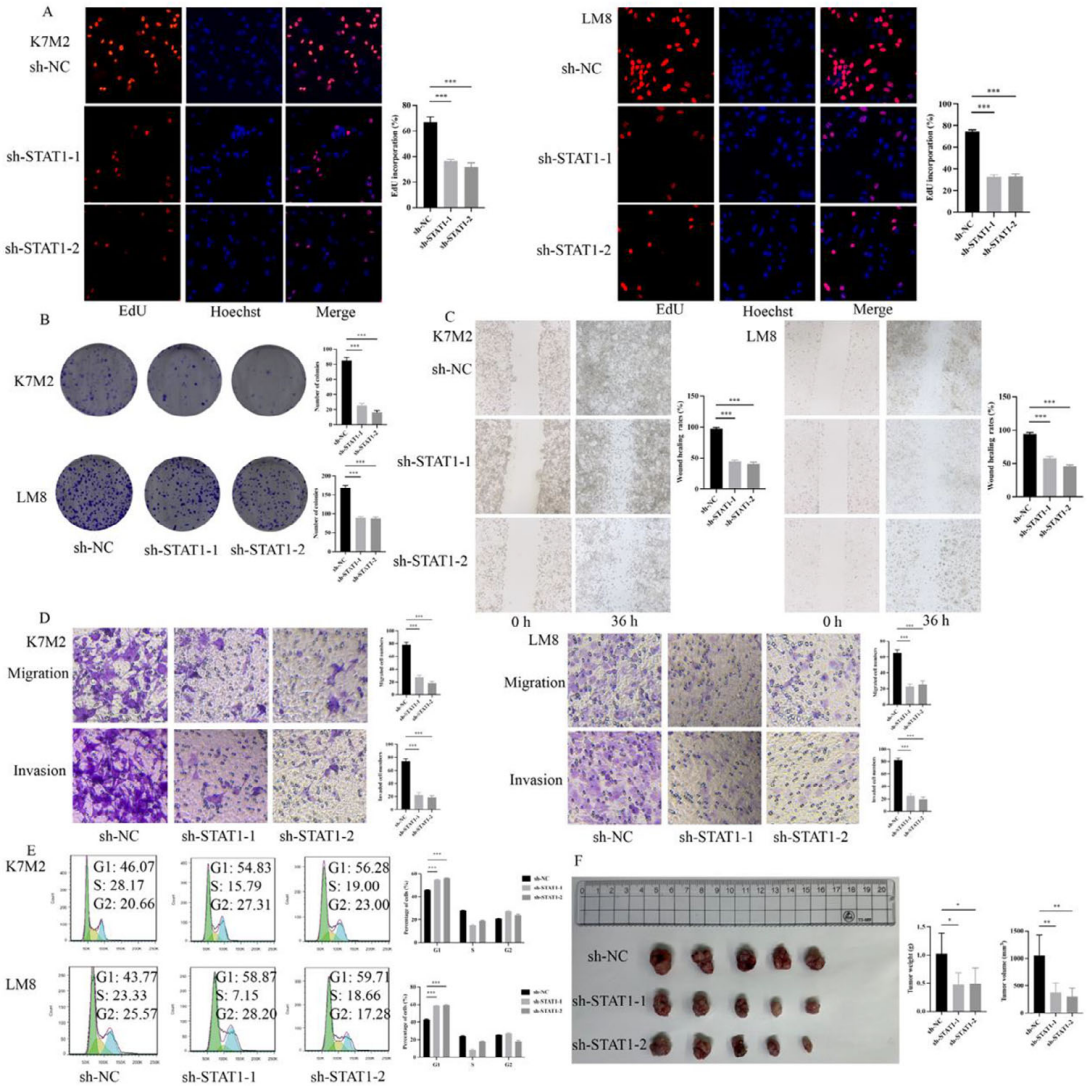
Effects of STAT1 knockdown on osteosarcoma cell proliferation and migration, invasion, cell cycle, and tumor growth in vivo. A) The EdU assay revealed that the proliferation capacity of K7M2 and LM8 cells in sh‐STAT1‐1 and sh‐STAT1‐2 group was significantly reduced compared with sh‐NC control group. B) Colony formation assays showed reduced colony formation in STAT1 knockdown K7M2 and LM8 cells compared to control group. C) Wound healing assays demonstrated reduced migration ability in STAT1 knockdown K7M2 and LM8 cells. D) Transwell assay showed decrease migration and invasion in STAT1 knockdown cells compared to control group. E) The proportion of G1 phase in K7M2 and LM8 cells with STAT1 knockdown was increased compared to sh‐NC group. F) Tumor growth was significantly inhibited in STAT1 knockdown groups in vivo. *P < 0.05, **P < 0.01, ***P < 0.001.

EdU and colony formation assays demonstrated that STAT1 knockdown significantly inhibit cell proliferation in sh‐STAT1‐1 and sh‐STAT1‐2 groups compared to the sh‐NC group (**Figure** **6**A,B). In addition, the wound healing and transwell assays revealed that downregulating STAT1 reduce the migration and invasion abilities in K7M2 and LM8 cells (Figure 6C,D). Cell cycle analysis indicated that STAT1 knockdown led to cell cycle arrest at the G1 phase (Figure 6E). The in vivo experiment showed that tumor weight and volume were obviously reduced in the sh‐STAT1‐1 or sh‐STAT1‐2 groups compared to the sh‐NC group (Figure 6F).

### 2.7 Combined the Overexpression of HTR2B and Knockdown of STAT1 can Further Suppress Proliferation, Migration, Invasion, and Arrests the Cell Cycle In Vitro, and Reduce Tumor Growth In Vivo

**Figure 7:**
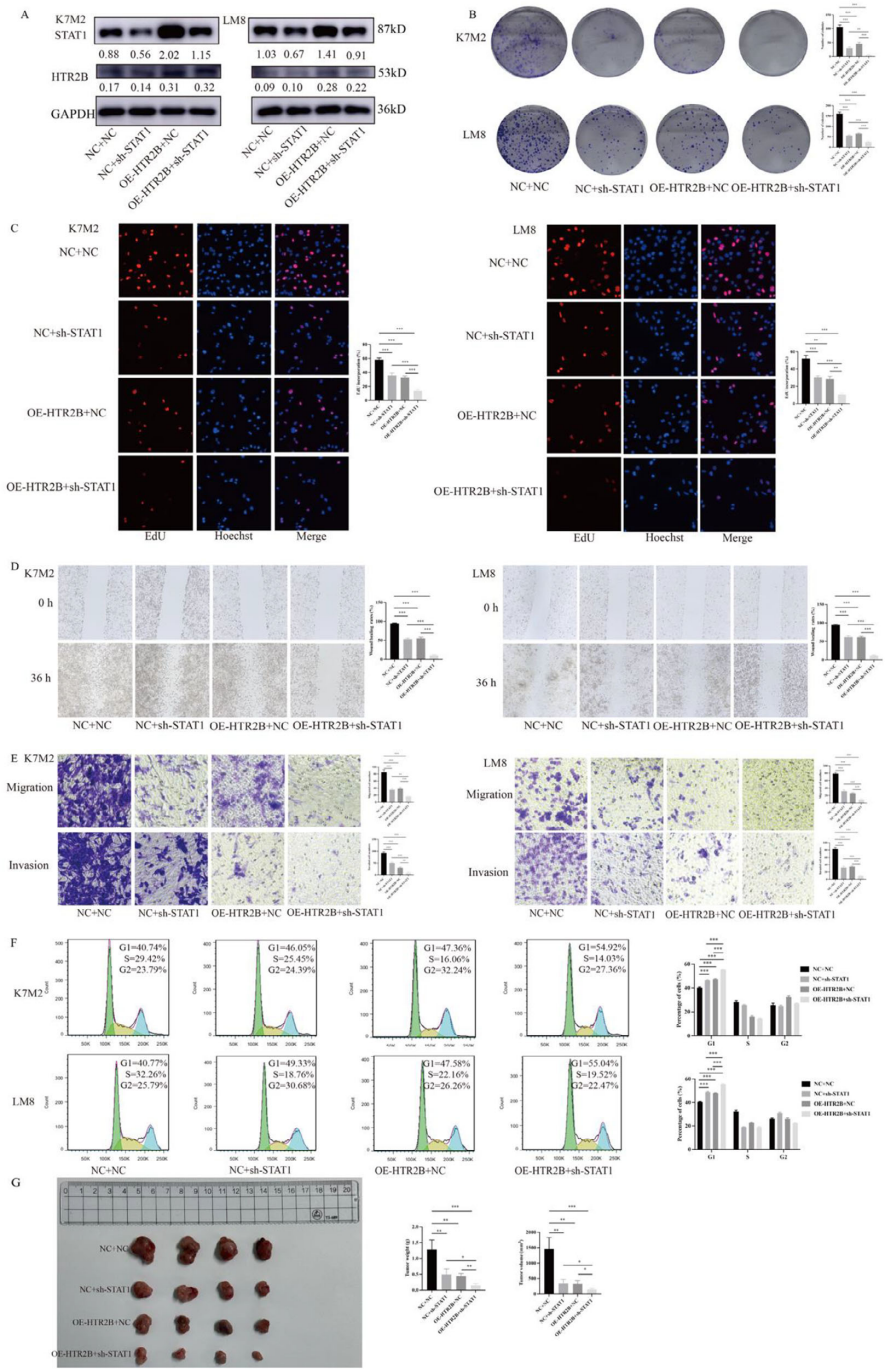
Effects of HTR2B overexpression combined with STAT1 knockdown on osteosarcoma cell proliferation and migration, invasion, cell cycle, and tumor growth in vivo. A) The western blot assays confirmed a transfected efficiency of OE‐HTR2B and sh‐STAT1 in K7M2 and LM8 cells. B) Colony formation assay found that colony formation ability further decreased in OE‐HTR2B combined with sh‐STAT1 group. C) The EdU assay revealed that OE‐HTR2B combined with sh‐STAT1 can further reduce cell proliferation ability in K7M2 and LM8 cells. D) Wound healing assay revealed that OE‐HTR2B combined with sh‐STAT1 can further suppress migration ability in K7M2 and LM8 cells. E) Transwell assay result demonstrated that OE‐HTR2B combined with sh‐STAT1 can further inhibit the migration and invasion abilities in K7M2 and LM8 cells. F) The cell cycle analysis showed that G1 phase is further arrested in OE‐HTR2B combined with sh‐STAT1 group in K7M2 and LM8 cells. G) The in vivo experiment revealed that OE‐HTR2B combined with sh‐STAT1 group can further suppress tumor growth. *P < 0.05, **P < 0.01, ***P < 0.001.

The western blot analysis confirmed the transfection efficacity of OE‐HTR2B and sh‐STAT1, as shown in **Figure** **7**A. The colony formation and EdU assays showed that cell proliferation ability was further blocked in OE‐HTR2B combined with sh‐STAT1 group in K7M2 and LM8 cells (Figure 7B,C). The wound healing and transwell assays found that OE‐HTR2B combined with sh‐STAT1can further reduce the migration and invasion abilities in K7M2 and LM8 cells (Figure 7D,E). Additionally, cell cycle detection demonstrated that OE‐HTR2B combined with sh‐STAT1can further arrest osteosarcoma cell cycle in G1 phase (Figure 7F). Moreover, the in vivo experiment confirmed that OE‐HTR2B combined with sh‐STAT1can further suppresses tumor growth in vivo (Figure 7G).

### 2.8 Activation of HTR2B and Knockdown of STAT1 Suppress Osteosarcoma Progression via Activating the NLRP3 Inflammasome

**Figure 8:**
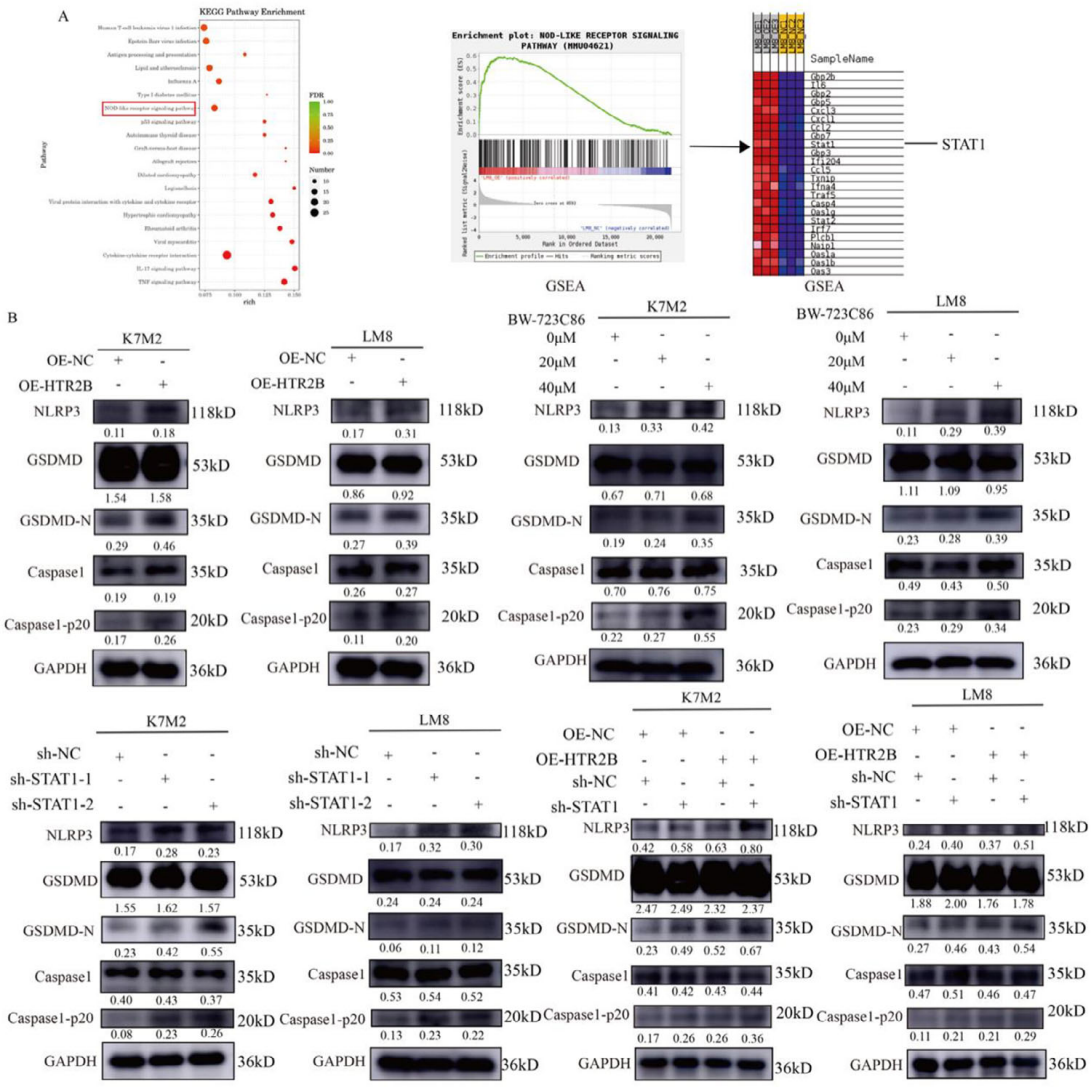
Activation of HTR2B suppresses osteosarcoma progression via NLRP3 inflammasome pathway. A) KEGG enrichment analysis of transcriptome sequencing showed that the NOD‐like receptor signaling pathway is enriched, which containing the STAT1. B) Western blot analysis showed that promotion of HTR2B protein by lentivirus or BW‐723C86, knockdown of STAT1, and HTR2B overexpression combined with STAT1 knockdown led to the activation of the NLRP3 inflammasome pathway.

Having confirmed the detrimental effect of HTR2B and STAT1 on osteosarcoma, we focused on discovering the underlying molecular mechanisms. The KEGG pathway enrichment of different expression genes in OE‐NC and OE‐HTR2B groups found that the NOD‐like receptor signaling pathway, and the Gene Set Enrichment Analysis (GSEA) enrichment analysis also enriched in the NOD‐like receptor signaling pathway (**Figure** **8**A). Moreover, we found that STAT1, NLRP3, GSDMD, and Caspase1 were enriched in NOD‐like receptor signaling pathway. Therefore, NLRP3‐inflammasome may be the underlying molecular mechanism of HTR2B in osteosarcoma. As shown in Figure 8B, the results of western blots showed that promoting HTR2B expression by lentivirus or BW‐723C86 can both activate NLRP3 inflammasome. Knockdown of STAT1 can also activate NLRP3 inflammasome. Moreover, Combined the overexpression of HTR2B and knockdown of STAT1 can further suppresses osteosarcoma progression via NLRP3 inflammasome. However, our study found that STAT1 expression is promoted after HTR2B activation, which suggests that increase the expression of STAT1 after HTR2B activation may be partially resistant to therapeutic values of HTR2B.

### 2.9 HTR2B Activation Modulates the Tumor Immune Microenvironment in Osteosarcoma

**Figure 9:**
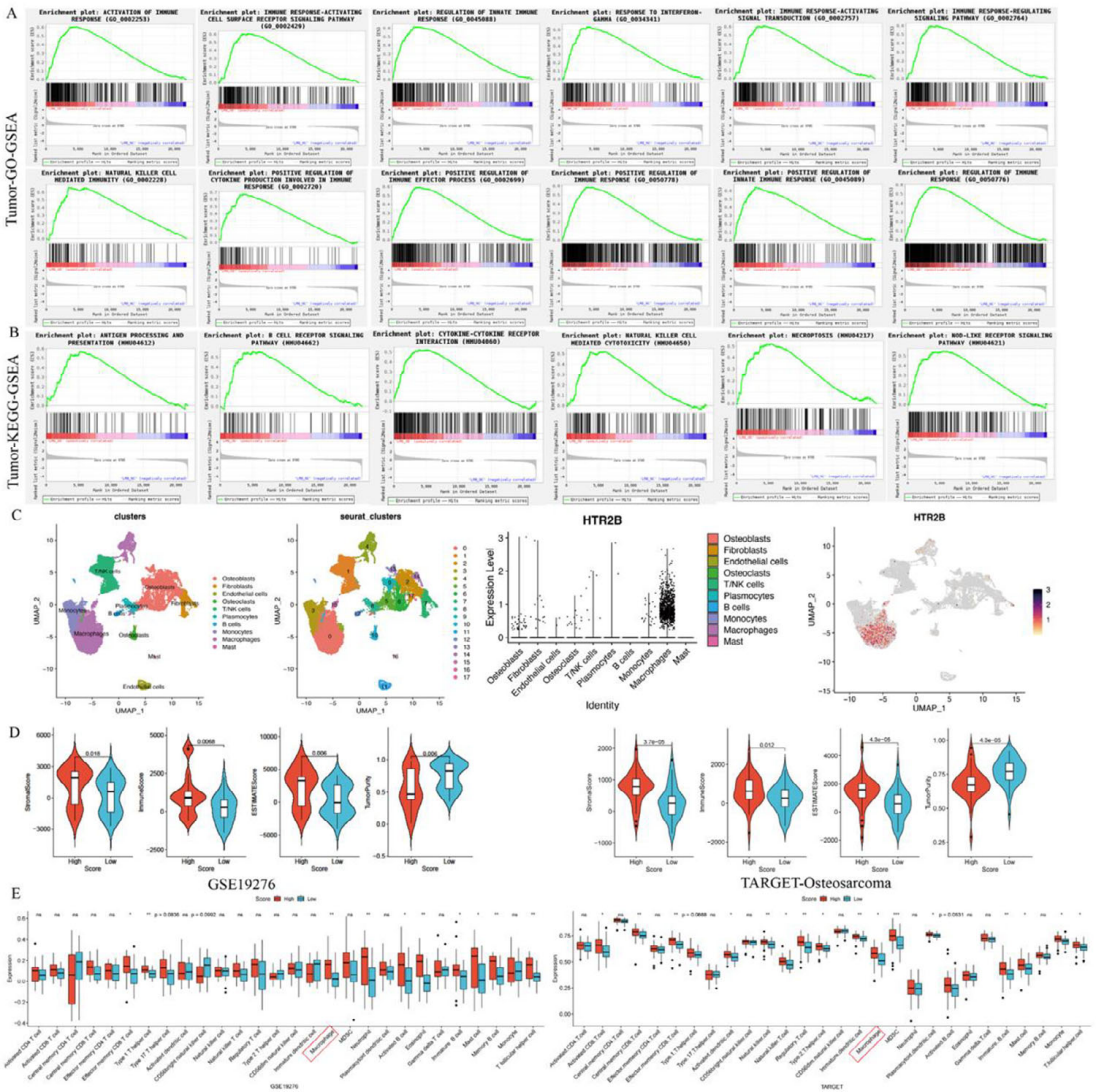
HTR2B activation and its impact on the tumor immune microenvironment. A) The GO‐GSEA analysis of tumor tissue transcriptome sequencing revealed that HTR2B activation associated with multiple immune reaction processes. B) The KEGG‐GSEA analysis of tumor tissue transcriptome sequencing found that activation of HTR2B associated with multiple immune reaction processes. C) The analysis of osteosarcoma single‐cell database revealed the enrichment of HTR2B in macrophage. D) The microenvironment scoring of HTR2B expression. E) Bioinformatics analysis of GSE19276 and TARGET‐Osteosarcoma databases showed that high HTR2B expression correlates with increased immune cell infiltration, containing macrophage. *P < 0.05, **P < 0.01.

RNA sequence analysis of LM8‐OE‐NC (NC) and LM8‐OE‐HTR2B (OE) tumor tissue revealed enrichment of immune‐related GO‐GSEA and KEGG‐GSEA signaling pathways (**Figure** **9**A,B). The immune cell infiltration analysis found that overexpression of HTR2B can promote multiples immune cells infiltration (Figure S5, Supporting Information). The analysis of Osteosarcoma single‐cell database showed the enrichment of HTR2B in macrophages (Figure 9C). Additionally, high expression of HTR2B associated with high StromalScore, ImmuneScore, and ESTIMATEScore, and low TumorPurify both in GSE19276 and TARGET‐Osteosarcoma databases (Figure 9D). Moreover, the relationships between HTR2B and immune cell analysis demonstrated that high expression of HTR2B correlated with elevated macrophage levels in the GSE19276 and TARGET‐Osteosarcoma databases (Figure 9E). Consequently, the impact of HTR2B on macrophage group in the tumor microenvironment was further investigated.

### 2.10 Single‐Cell Sequencing Reveals HTR2B Activation Promotes OASL1+ Macrophage Production

**Figure 10:**
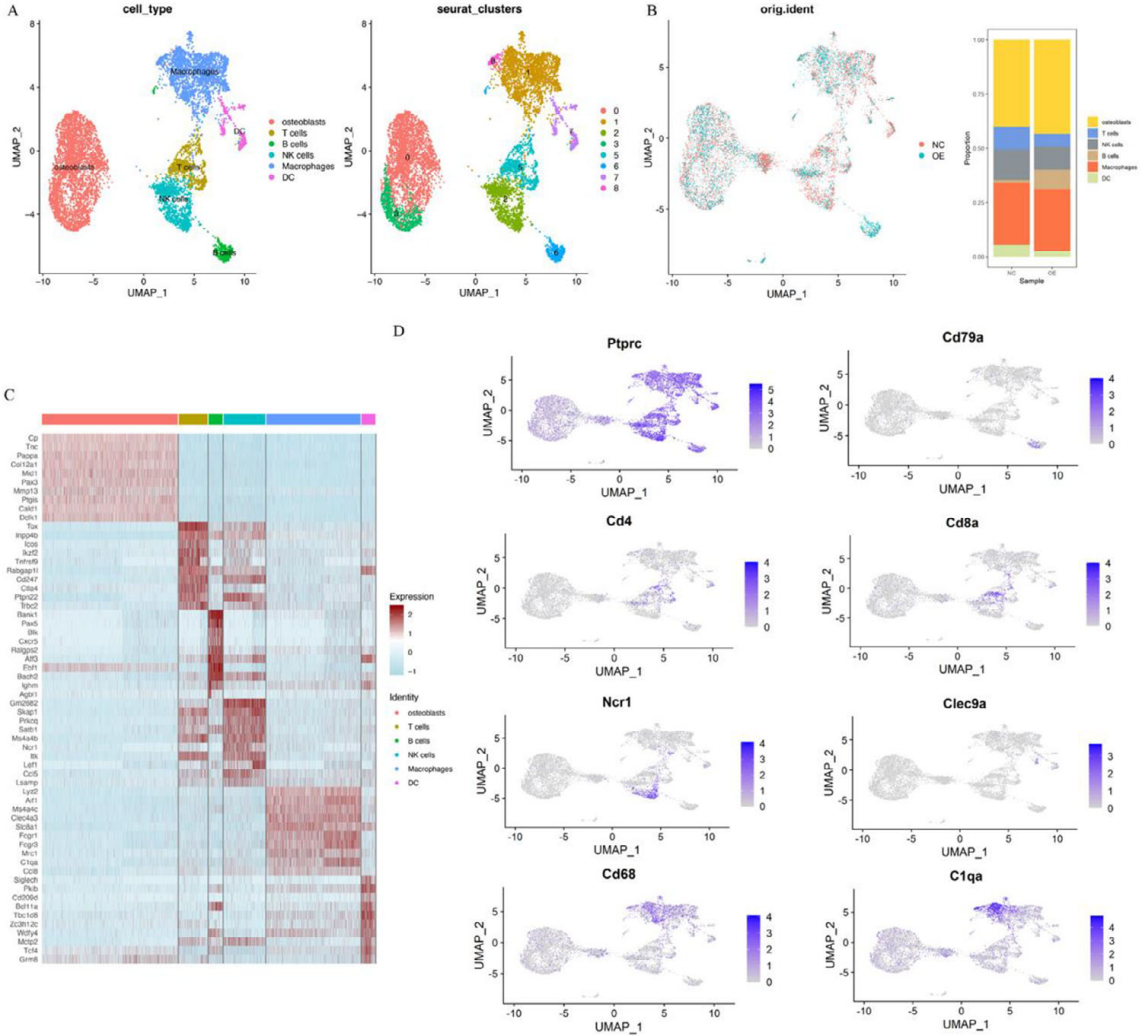
Single‐cell sequencing results and marker enrichment. A) Unsupervised clustering analysis identified 6 distinct cells into Osteoblasts, T cells, B cells, NK cells, Macrophages, and DC clusters, respectively. B) The distribution and percentage of each subgroups in NC and OE group; C) The markers heatmap of different clusters in single cell. D) The representative genes enriched in T cells, B cells, NK cells, Macrophages, DC clusters, respectively.

**Figure 11:**
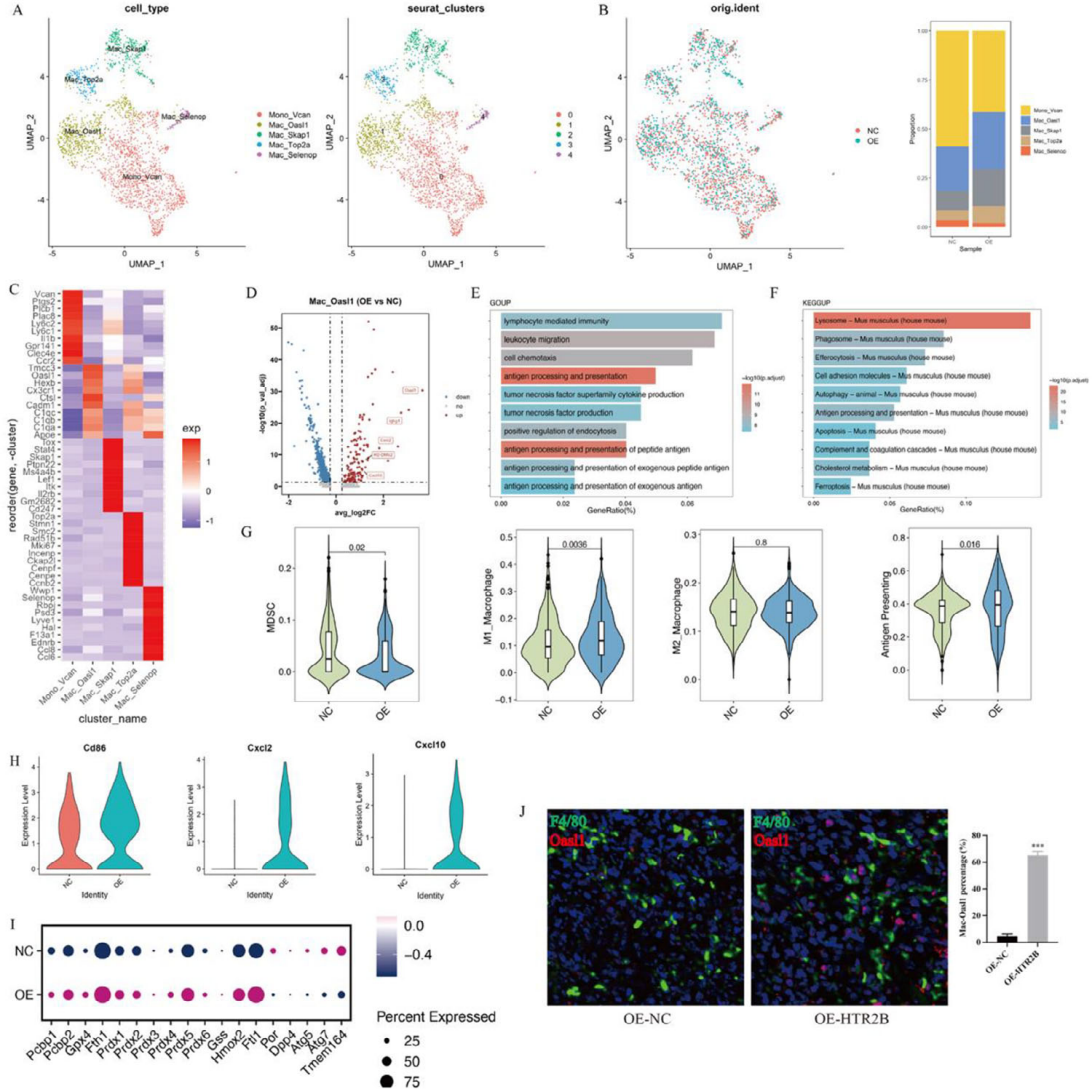
Macrophage subgroup annotations and potential functions. A) The macrophage subgroup has been defined as Mono‐Vcan, Mac‐Oasl1, Mac‐Skap1, Mac‐Top2a, and Mac‐Selenop clusters. B) The distribution and percentage of each subgroup in NC and OE group. C) Heatmap of markers for macrophage subgroups. D) The volcano map of differential genes expression in Mac‐Oasl1 subgroup. E) GO enrichment analysis found that signaling pathways related to antigen presentation were significantly enriched. F) KEGG enrichment analysis revealed that antigen processing and presentation, and ferroptosis pathway were enriched. G) Scoring analysis showed decreased MDSC and increased M1 macrophage and antigen‐presenting scores in the OE group, with unchanged M2 macrophage scores. H) Costimulatory molecules analysis showed that CD86, Cxcl2, and Cxcl10 increased in OE group, which could increase immune infiltration. I) Ferroptosis‐related gene expression indicated reduced ferroptosis in the Oasl1 subgroup, indicating that the Oasl1 subgroup was resistant to ferroptosis. J) The multiple immunofluorescence staining results confirmed that OASL1+macrophages is promoted in OE‐HTR2B group. ***P < 0.001.

To investigate the specific effect of HTR2B activation in macrophage, we examined the single‐cell transcriptomes of immune cells in LM8‐OE‐NC (NC) and LM8‐OE‐HTR2B (OE) tumor tissue. Unsupervised clustering analysis identified 6 distinct cell clusters (Osteoblasts, T cells, B cells, NK cells, Macrophages, DC), respectively (**Figure** **10**A,B). Cell clusters marker heatmap of different groups were identified by cell‐specific marker expression (Figure 10C,D). Within the macrophage cluster, subgroups were annotated as Mono‐Vcan, Mac‐Oasl1, Mac‐Skap1, Mac‐Top2a, and Mac‐Selenop (**Figure** **11**A,B). Cell clusters marker heatmap of macrophage subgroups were identified by cell‐specific marker expression (Figure 11C). Additionally, the Mac‐Oasl1 subgroup exhibited a significant upregulation of the Oasl1, Cxcl2, and Cxcl10 genes (Figure 11D). The GO enrichment analysis of the Mac‐Oasl1 subgroup identified enrichment in antigen processing and presentation, antigen processing and presentation of peptide antigen, tumor necrosis factor superfamily cytokine production, and tumor necrosis factor production are enriched (Figure 11E). The KEGG enrichment analysis highlighted in antigen processing and presentation and ferroptosis (Figure 11F). Scoring of macrophage subgroups revealed decreased MDSC scores and increased M1 macrophage and antigen‐presenting scores in the LM8‐OE‐HTR2B (OE) group, with unchanged M2 macrophage scores (Figure 11G). Costimulatory molecule analysis showed that CD86, Cxcl2, and Cxcl10 increased in the LM8‐OE‐HTR2B (OE) group, which could enhance immune infiltration (Figure 11H). Further comparison of ferroptosis‐related genes between groups showed that the expression of ferroptosis‐promoting genes was decreased and ferroptosis‐inhibiting genes increased, indicating that the Mac‐Oasl1 subgroup was resistant to ferroptosis (Figure 11I). The Multiplex Immunofluorescence (mIF) staining of tumor tissues confirmed that overexpression of HTR2B promote the production of Oasl1+macrophage (Figure 11J).

**Figure 12:**
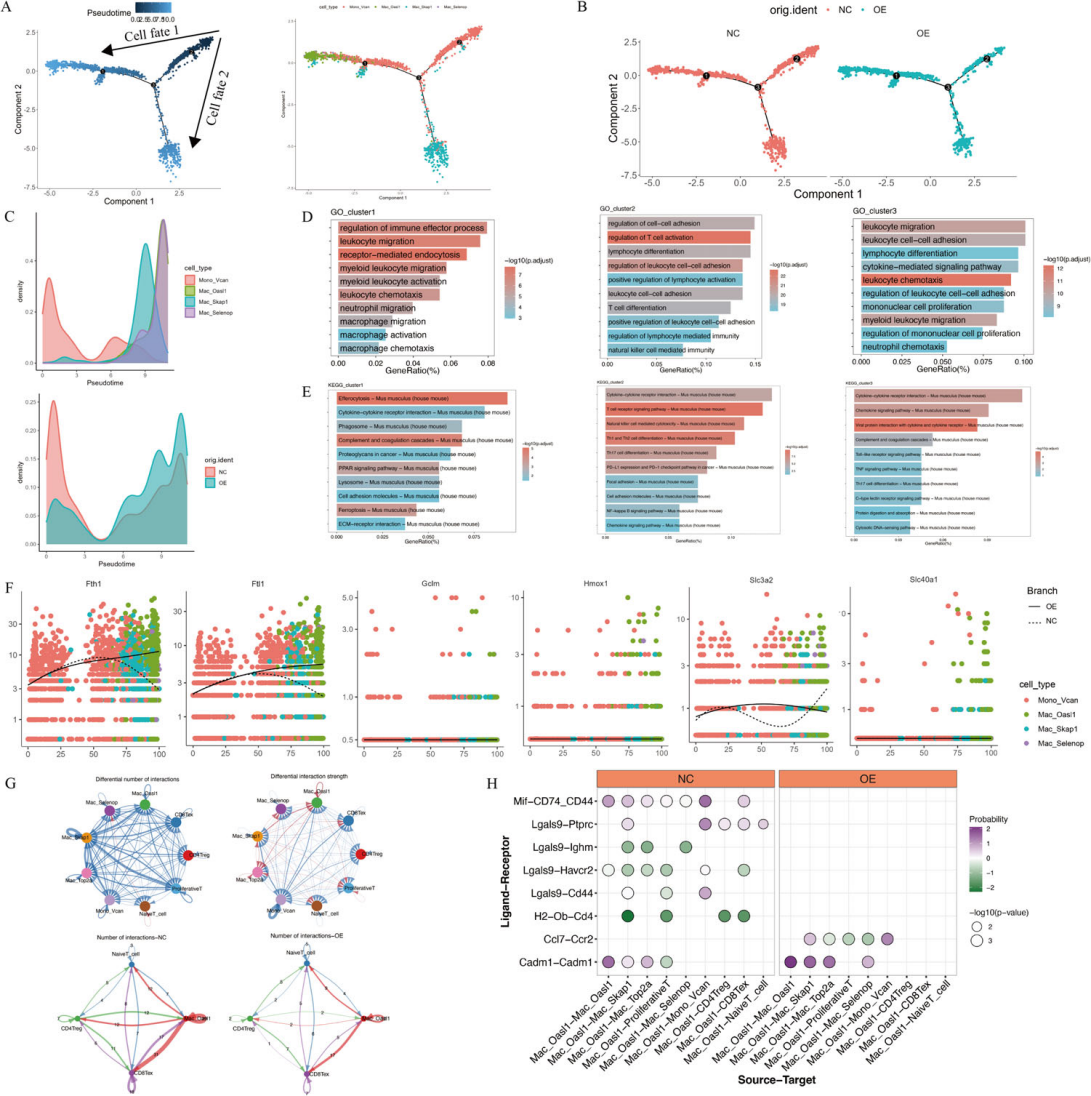
Pseudotime analysis of macrophage subgroups and cell communications. A) Unsupervised trajectory of macrophage transition along pseudotime using Monocle. B) The monocle facet in NC and OE group. C) The cluster and group density transition along pseudotime. D) GO enrichment analysis showed that cluster1 subgroup was associated with macrophage activation and leukocyte activation, cluster2 subgroup is associated with T cell activation and differentiation, and cluster3 subgroup is associated with leukocyte activation and chemotaxis. E) KEGG enrichment analysis showed that cluster1 subgroup was associated with ferroptosis, the cluster2 subgroup is associated with of T cell receptors and subtypes differentiation, the cluster3 subgroup is associated with cytokine receptors and chemokines. F) The ferroptosis enrichment pathway in cluster1 subgroup showed that the expression levels of ferroptosis suppressor genes fth1 and ftl1 gradually increased in Oasl1 subgroup. G) Cell communication analysis showed enhanced interactions among macrophage subtypes and weakened interactions with CD8 Tex and CD4 Treg. H) Ligand receptor analysis showed that Mac‐Oasl1subgroup macrophages may enhance the interaction between giant cells through Cadm1‐Cadm1 and Ccl7‐Ccr2, while the expressions of Mif‐CD74‐ CD44 (macrophage migration inhibitor) and Lgals9‐Havcr2 were significantly inhibited in OE‐HTR2B group, the interaction with CD8 Tex and CD4 Treg may be weakened by Lgals9‐Ptprc and H2‐Ob‐ CD4.

Dynamic analysis along pseudotime showed distinct roles for macrophage subgroups (**Figure** **12**A–C). The GO enrichment analysis showed that the cluster1 subgroup was associated with macrophage activation and leukocyte activation, the cluster2 subgroup was related to T cell activation and differentiation, and the cluster3 subgroup was associated with leukocyte activation and chemotaxis (Figure 12D). The KEGG enrichment analysis indicated that the cluster1 subgroup was correlated with ferroptosis, the cluster2 subgroup was associated with of T cell receptors and subtypes differentiation, the cluster3 subgroup was related to cytokine receptors and chemokines (Figure 12E). Further analysis of ferroptosis enrichment pathway revealed increased expression of ferroptosis suppressors (Fth1 and Ftl1) in the Mac‐Oasl1 subgroup (Figure 12F). The cell communications between macrophages and T cells showed that Mac‐Oasl1enhanced their interaction with other subtypes of macrophages, but reduced their interaction with CD8 Tex and CD4 Treg cells in Mac‐Oasl1 subgroup (Figure 12G). Ligand–receptor analysis indicated that Mac‐Oasl1subgroup might enhance the interaction between other macrophages through Cadm1‐Cadm1 and Ccl7‐Ccr2, while the expressions of Mif‐CD74‐CD44 (macrophage migration inhibitor) and Lgals9‐Havcr2 were significantly inhibited in LM8‐OE‐HTR2B (OE) group, and the interaction with CD8 Tex and CD4 Treg may be weakened by Lgals9‐Ptprc and H2‐Ob‐ CD4 (Figure 12H). Additionally, the classification diagrams of T cells, B cells, NK cells, DC types found that overexpression of HTR2B can enhance antitumor immunity in osteosarcoma tumor microenvironment (Figure S6, Supporting Information).

### 2.11 RAW264.7‐Oasl1 Suppresses Proliferation, Migration, and Invasion, Arrests the Cell Cycle of Osteosarcoma In Vitro

To further investigate the effects of Mac‐Oasl1 on osteosarcoma. Oasl1 overexpression plasmid was used to transfect RAW264.7 cells, and then indirectly culture with K7M2 and LM8 cells. The RT‐qPCR analysis confirmed that Oasl1 is significantly overexpressed in RAW264.7‐Oasl1 compared to RAW264.7‐Vector (Figure S7A, Supporting Information). The colony formation and EdU assays showed that cell proliferation ability is blocked in K7M2 and LM8 cells when cocultured with RAW264.7‐Oasl1 cell (Figure S7B,C, Supporting Information). The wound healing and transwell assays indicated that cocultured with RAW264.7‐Oasl1 cell can reduce the migration and invasion abilities in K7M2 and LM8 cells (Figure S7D,E, Supporting Information). Additionally, cell cycle detection demonstrated that cocultured with RAW264.7‐Oasl1 cell can arrest the osteosarcoma cell cycle in the G1 phase (Figure S7F, Supporting Information).

## 3 Discussion

The combination of surgery and chemotherapy has significantly improved survival rates for osteosarcoma patients over the past 40 years, increasing from 20% to nearly 70%.^[^ ^22^, ^23^ ^]^ However, the survival rates in recurrence and metastasis patients are far from satisfactory.^[^ ^24^, ^25^ ^]^ Accumulating studies confirmed that antitumor therapy targeting serotonin receptors may be the possibility of curing the tumor.^[^ ^11^, ^26^ ^]^ Exploring the potential therapeutic target is significant in overcoming the poor survival outcomes in osteosarcoma patients. Our study identified HTR2B as a potential prognostic biomarker and explores its role in osteosarcoma progression, offering new insights into its therapeutic potential. HTR2B belongs to the serotonin receptor family, and participates in tumor progression.^[^ ^27^ ^]^ A previous study has shown that HTR2B is downregulated in aggressive breast cancer types, correlating with poor overall survival.^[^ ^20^ ^]^ In colitis‐associated cancer, HTR2B knockout mice exhibited increased colitis and tumor severity, supporting its role as a potential tumor suppressor.^[^ ^14^ ^]^ These studies align with our study, which demonstrates that HTR2B activation can suppress osteosarcoma progression.

Our study reveals that HTR2B activation promotes STAT1 expression. The GSEA enrichment analysis found that cellular response to interferon‐gamma pathway is enriched. Previous studies confirmed that the STAT1 protein can be promoted through the activation of classical interferon‐gamma signaling pathway.^[^ ^28^, ^29^, ^30^ ^]^ However, low STAT1 expression correlates with lower proliferation, migration, invasion, and tumor growth, suggesting that the overexpression of STAT1 after HTR2B activation might undermine therapeutic efficacy. STAT1 is an essential protein that connects cell membrane receptors and effectors for signal transduction.^[^ ^31^, ^32^ ^]^ Under the stimulation of extracellular signals, STAT1 in the cytoplasm can be polymerized to form homologous or heterodimers, and then enter the nucleus to promote the transcription of target genes.^[^ ^33^, ^34^ ^]^ It has been reported that the activation of STAT1 can promote the apoptosis of tumor cells and thus play an antitumor role.^[^ ^35^ ^]^ However, overactivation of STAT1 in some tumors does not induce tumor cell apoptosis, but promotes tumor cell proliferation.^[^ ^36^, ^37^ ^]^ In some cancers, such as breast and lung cancer, overexpression of STAT1 is associated with resistance to ionizing radiation and chemotherapy.^[^ ^38^, ^39^ ^]^ In colon cancer, STAT1 has been shown to have a prosurvival role in cancer cells having mutated K‐ras genes, and a chemical probe inhibitor targeting STAT1 inhibits the properties of stemness and angiogenesis in cell lines.^[^ ^40^, ^41^ ^]^ In glioblastoma, high STAT1 expression promotes a protumorigenic phenotype by the Wnt/βcatenin both in vitro and in vivo.^[^ ^42^ ^]^ These studies confirmed the cancer‐driver role of STAT1 in multiple types of cancers. Our findings indicate that HTR2B activation increases STAT1 expression, the subsequent high STAT1 levels might limit the therapeutic benefits of HTR2B. The regulation of HTR2B in NLRP3 inflammasome pathway in cancer has not been reported in previous studies. KEGG pathway analysis and GSEA revealed significant enrichment in the NOD‐like receptor signaling pathway, with STAT1 also being a key component. In ovarian cancer, the activation of NLRP3‐induced pyroptosis can inhibit ovarian cancer progression.^[^ ^43^ ^]^ In hepatocellular carcinoma cells, Shuanghua decoction exerts anticancer activity by activating NLRP3 inflammasome.^[^ ^44^ ^]^ In cervical cancer, targeting Foxm1 mediates the activation of NLRP3 inflammasome and stimulates CD8+ T cell antitumor immunity.^[^ ^45^ ^]^ Our results suggest that HTR2B might influence osteosarcoma progression through the NLRP3 inflammasome pathway.

HTR2B is closely related to the regulation of tumor microenvironment.^[^ ^46^ ^]^ Related studies have found that HTR2B positively correlates with the infiltration of CD8+ T cells, macrophages, and other immune cells in the tumor microenvironment during the progression of breast cancer and gastric cancer.^[^ ^47^ ^]^ Our study investigates the impact of HTR2B on the tumor microenvironment, and found that activation of HTR2B can promote OASL1+ macrophage production to enhance antitumor immunity through multiple pathways. Our single‐cell analysis shows that ferroptosis‐related genes are suppressed in OASL1+ macrophages in the OE‐HTR2B group. Ferroptosis is a unique form of cell death that relies primarily on accumulating irons and lipid peroxides within the cell.^[^ ^48^ ^]^ Ferroptosis is closely related to the immune microenvironment.^[^ ^49^ ^]^ In the tumor microenvironment, tumor‐associated macrophages mainly exhibit immunosuppressive M2 phenotype, which is sensitive to ferroptosis, while M1‐type macrophages are resistant to ferroptosis.^[^ ^50^ ^]^ In addition, the cell communications analysis showed that OASL1+ macrophage enhanced their interaction with other subtypes of macrophages, but weakened their interaction with CD8 Tex and CD4 Treg. CD8 Tex and CD4 Treg mainly act as immunosuppressive in the tumor immune microenvironment. A study unveiled differential CD8 Tex cells within the osteosarcoma microenvironment which found that patients in the low CD8 Tex score group demonstrated a more favorable prognosis, increased immune cell infiltration, and heightened responsiveness to treatment compared to those in the high CD8 Tex score group.^[^ ^51^ ^]^ The promoting infiltration of CD4 Treg cells predicts poor prognosis in osteosarcoma patients.^[^ ^52^ ^]^ This suggests that HTR2B activation could modulate the immune landscape to enhance antitumor responses.

## 4 Conclusions

In summary, our study identifies HTR2B as a critical tumor suppressor in osteosarcoma progression, functioning through the NLRP3 inflammasome pathway. However, STAT1 may partially counteract the tumor‐suppressive effects of HTR2B. Furthermore, the activation of HTR2B enhances the production of OASL1+ macrophages, thereby boosting antitumor immunity in vivo. These findings underscore HTR2B's potential as a therapeutic target and provide insights into its role in modulating the immune microenvironment of osteosarcoma.

## 5 Experimental Section

### Data Acquisition and Processing

Gene expression profiles for osteosarcoma were downloaded from TARGET‐Osteosarcoma from the TARGET dataset (https://ocg.cancer.gov/programs/target) and the GSE19276 dataset from the GEO Gene Expression Data set (https://www.ncbi.nlm.nih.gov/geo/). Subsequent bioinformatics analysis was conducted using R software.

### Cell Culture

The K7M2 cell line was purchased from iCellbioscience (Shanghai, China), and the LM8 cell line was obtained from Haixing Biosciences (Suzhou, China). RAW264.7 cell line was purchased from ShuochengBio (Shanghai, China). All cell lines were cultured in high‐glucose Dulbecco's modified Eagle' s medium (DMEM; Viva Cell Bioscience, Shanghai) supplemented with 10% fetal bovine serum (FBS; Viva Cell Bioscience, Israel) and 1% penicillin/streptomycin (Viva Cell Bioscience, Shanghai). Cells were maintained under standard conditions at 37 °C in a humidified atmosphere with 5% CO₂.

### Lentivirus Transfection

Lentivirus for HTR2B overexpression and STAT1 knockdown were purchased from Gemma Gene (Suzhou, China). A density of 5 × 10^−4^ cells per well was seeded in six‐well plates and allowed to adhere overnight. The following day, cells were transfected with the respective lentiviruses according to the manufacturer's introductions. The efficiency of transfection was assessed by measuring HTR2B and STAT1 mRNA and protein expression levels.

### Plasmid Transfection

The Vector control and Oasl1 overexpression plasmids were purchased from MiaoLingPlasmid (Shanghai, China). RAW264.7 cells were seeded in 6‐well plates at a density of 5 × 10^−^⁵ cells per well and allowed to adhere overnight. When the cell density reached 70%–80%, the Vector control or Oasl1 overexpression plasmid was transfected using the Lipo8000 (Beyotime, China). The transfected efficiency was confirmed by RT‐qPCR.

### RT‐qPCR

Total RNA was extracted from cells using the RNA‐Quick Purification Kit (ShareBio, China). ABScript II cDNA First Strand Synthesis Kit (ABclonal, China) was used to reverse RNA to cDNA, and the amplification of cDNA was examined on QuantStudio 1 (Thermo Fisher Scientific Inc, USA) by using Taq SYBR Green qPCR Premix Kit (iScience, China). Primers were synthesized by Sangon Biotech (Shanghai, China), with the following sequences: HTR2B, Forward: TCTTCGCACCTCTCACCATCATG, Reverse: GGCACAGTCCACCGTGTTAGG, STAT1, Forward: TGCTGTGCCTCTGGAATGATGG, Reverse: CCTGGCTGCTGGTCCTTGAG, Oasl1, Forward: AGGTAGGCTGCTTTGGGAATGG, Reverse: CCTGATGGTGCITGGCTTCTTC, GAPDH, Forward: AGAAGGTGGTGAAGCAGGCATC, Reverse: CGAAGGTGGAAGAGTGGGAGTTG. GAPDH was used as the internal control to normalize gene expression levels.

### Cell Counting Kit‐8 Assay

A density of 1*10‐3 cells was seeded in 96 well plates and incubated overnight. The next day, cells were incubated with different concentrations of BW‐723C86, and cultured for 72 h. Then, the medium replaced by 100 µl fresh medium which containing 10 µl CCK‐8 solution (New Cell & Molecular Biotech Co., Ltd, China) and incubated at 37 °C for 2 h. Absorbance values were measured at 450 nm using a microplate reader.

### 5‐ethynyl‐2′‐deoxyuridine (EdU) Assay

A density of 5 × 10^−4^ cells was seeded in 24 well plates and incubated overnight. In the lentivirus group, the culture medium was replaced with a fresh medium, and cells were cultured for an additional 24 h. In the BW‐723C86 (MedChemExpress, USA) treatment group, cells were cultured for 24 h in a medium containing different concentrations of BW‐723C86 (0, 20, and 40 µm). In the RS‐127445 treatment group, cells were cultured for 24 h in a medium containing RS‐127445. In the coculture treatment group, transwell chambers were placed in 24 well plates, and transfected RAW264.7 cells were added to the upper chamber and incubated for 24 h. Then, cells were incubated with EdU (10 µm) working solution for 2 h (RS‐127445 treatment group for 1 h) at 37 °C. After incubation, cells were fixed with 4% polyformaldehyde for 15 min and permeabilized with 3% Triton X‐100 in PBS for 15 min. The cells were then incubated with the click reaction solution at room temperature for 30 min in the dark. Nuclei were stained with 1X Hoechst 33 342, and the EdU incorporation was visualized and assessed under a fluorescence microscope.

### Colony Formation Assay

A density of 800 cells was seeded in six‐well plates and incubated overnight. For the lentiviral transfection group, cells were cultured in a medium for 6 days. For the BW‐723C86 treatment group, cells were cultured in a medium containing different concentrations (0, 20, and 40 µm) of BW‐723C86 for 6 days. For the RS‐127445 (MedChemExpress, USA) treatment group, cells were cultured in a medium containing of RS‐127445 for 6 days. For the coculture group, transwell chambers were placed in six‐well plates to separate RAW264.7‐Vector or RAW264.7‐Oasl1 and osteosarcoma cells, and cocultured for 6 days. After the incubation, cells were fixed with 4% polyformaldehyde for 15 min and stained with 0.1% crystal violet (Solarbio, Beijing, China) for 20 min. Colonies containing more than 50 cells were counted and recorded.

### Wound Healing Assay

A density of 5 × 10^−5^ cells was seeded in six‐well plates and incubated overnight. When the cell density is close to 90%, Mitomycin C (1 µg mL^−1^, MedChemExpress, USA) was used to culture cells with 1 h. In the lentivirus transfection group, a sterile 200 µL pipette tip was used to create a scratch in the cell monolayer. In the BW‐723C86 treatment group, after creating the scratch with a sterile 200 µL pipette tip, then cells were cultured in a medium containing different concentrations of BW‐723C86 (0, 20, and 40 µm). In the RS‐127445 treatment group, after creating the scratch with a sterile 200 µL pipette tip, cells were cultured in a medium containing of (Dimethyl sulfoxide) DMSO or RS‐127445. In the coculture treatment group, after creating the scratch with a sterile 200 µL pipette tip, transwell chambers were placed in six‐well plates, and transfected RAW264.7 cells were added in the upper chamber for incubation. The wound healing results were photographed at 0 and 36 h (24 h in the RS‐127445 treatment group).

### Transwell Assay

Cell migration was assessed using a transwell chamber with an 8 µm pore size, while invasion was evaluated using Matrigel‐coated chambers (BD Biosciences, USA). In the lentivirus group, a density of 5 × 10^−4^ cells were seeded in the upper chamber, with 200 µL medium without FBS, with 600 µL of completed medium in the lower chamber. In the BW‐723C86 treatment group, cells were pretreated with different concentrations of BW‐723C86 (0, 20, and 40 µm) for 48 h prior to being seeded at 5 × 10^−4^ cells per well in the upper chamber with 200 µL medium without FBS, and 600 µL completed medium in the lower chamber. In the RS‐127445 treatment group, cells were pretreated with RS‐127445 for 48 h prior to being seeded at 5 × 10^−4^ cells per well in the upper chamber with 200 µL medium without FBS, and 600 µL completed medium in the lower chamber. In the coculture treatment group, a density of 5 × 10^−4^ K7M2 or LM8 cells were seeded in the upper chamber with 200 µL medium without FBS, and the transfected RAW264.7 cells were added in the lower chamber with 600 µL completed medium for incubation. After 24 h of incubation, cells were fixed with 4% polyformaldehyde for 15 min, and stained with 0.1% crystal violet (Solarbio, Beijing, China) for 20 min. The migrated and invaded cell numbers were quantified under a microscope.

### Flow Cytometry Assay

Cell cycle distribution was evaluated by flow cytometry assay. For cell cycle assessment, a density of 2 × 10^−5^ cells were seeded in six‐well plates and cultured overnight. In the lentivirus group, the culture medium was replaced with a fresh medium, and cells were cultured for an additional 24 h. In the BW‐723C86 treatment group, cells were cultured for 24 h in a medium containing different concentrations of BW‐723C86 (0, 20, and 40 µm). In the RS‐127445 treatment group, cells were cultured for 24 h in a medium containing RS‐127445. In the coculture treatment group, transwell chambers were placed in six‐well plates, and transfected RAW264.7 cells were added to the upper chamber for incubation 24 h. Cell cycle distribution was analyzed using the Cell Cycle and Apoptosis Analysis Kit (Beyotime, China).

### RNA Sequence Analysis

LM8‐OE‐NC and LM8‐OE‐HTR2B cells and tumors were subjected to RNA sequence analysis. Sequencing data were analyzed with the assistance of Bioprofile (Shanghai, China). Differential gene expression was analyzed using DESeq, with differentially expressed genes identified based on a |log2FoldChange| > 1 and a *P*‐value < 0.05. Volcano plots and heat maps of differentially expressed genes were generated using the ggplot2 package in R software. Bidirectional hierarchical clustering of differentially expressed genes and samples across all comparison groups was performed using the heatmap package in R software. Gene Ontology (GO) enrichment analysis was conducted using TopGO, while Kyoto Encyclopedia of Genes and Genomes (KEGG) pathway enrichment and were performed to further investigate the functional implications of differential gene expression.

### Tissue Microarray and Immunohistochemistry (IHC)

A human osteosarcoma tissue microarray, comprising 14 osteosarcoma samples, was manufactured in the hospital to analyze HTR2B expression and its correlation with patient prognosis. The experiment was permitted by the Ethics Committee of Shanghai Tenth People's Hospital (2020‐KN42‐01). Immunohistochemical staining for HTR2B was performed following the protocol provided with the Maxvision2 HRP‐Polymer anti‐Mouse/Rabbit IHC Kit (MXB, Biotechnologies). The staining results were scored from 0 to 3 according to the staining intensity of osteosarcoma cells (0, negative; 1, weakly positive; 2, moderately positive; 3, strongly positive). The mean percentage of positive staining osteosarcoma cells was also scored from 1 to 3 (1, <25%; 2, 25%–75%; 3, >75%). The final scores were the grade score multiplied by the percentage score, giving final scores of 0, 1, 2, 3, 4, 6, 9. Scores greater than 6 or 9 were considered as high expression, 3 or 4 considered as moderate expression, 1 or 2 considered as low expression, and 0 considered as negative expression.^[^ ^53^ ^]^

### Multiplex Immunofluorescence (mIF)

Tumor tissues from OE‐NC and OE‐HTR2B groups were subjected to mIF staining. Briefly, the slides were deparaffinized, hydrated, antigen retrieval, and endogenous peroxidase blocking. Then, the slides were incubated with primary antibodies against F4/80 (1:500, HUABIO) and Oasl1 (1:500, Bioss ANTIBODIES), and the suitable secondary antibody, and visualized using the tyramide signal amplification (TSA) technique. Nuclei acids were stained with 4′,6‐ diamidino‐2‐phenylindole (DAPI). The percentage of positively staining cells among all nucleated cells was recorded for further analysis.

### Western Blotting

Cell proteins were extracted using the Membrane protein, nuclear protein and cytoplasmic protein extraction kit (KeyGEN BioTECH, China). Protein quantification was determined using the Omni‐Easy Ready‐to‐use BCA Protein Assay Kit (EpiZyme, China). Protein samples (20 µg) were electrophoresed on 8% or 10% sodium dodecyl sulfate‐polyacrylamide gel for 80 min, and then transformed to polyvinylidene fluoride (PVDF) membranes (Millipore, USA) using NcmBlot Rapid Transfer Buffer (New Cell & Molecular Biotech Co., Ltd, China). Membranes were blocked with NcmBlot Blocking Buffer (New Cell & Molecular Biotech Co., Ltd, China) for 30 min at room temperature, and subsequently incubated overnight at 4 °C with primary antibodies against HTR2B (1:2000, Bioss ANTIBODIES), STAT1(1:1000, HUABIO Biotech), NLRP3 (1:1000, Abcam), GSDMD (1:1000, HUABIO), Caspase1 (1:5000, Proteintech), GAPDH (1:20 000, HUABIO) at 4 °C. The following day, the membranes were incubated with HRP Conjugated Goat anti‐Rabbit IgG polyclonal Antibody (1:30 000, HUABIO). The blots detection was evaluated using the FluorChem R detection system (ProteinSimple) with the affinity ECL Kit (Affinity, USA).

### In Vivo Experiment

Murine xenograft models were established in C3H mice (Shanghai SLAC Laboratory Animals Co., Ltd., Shanghai, China) for both lentivirus transfection, BW‐723C86 and RS‐127445 treatment groups, and permitted by Ethics Committee of Shanghai Tenth People's Hospital (SHDSYY‐2020‐3018). In the lentivirus transfection group, mice were divided into nine groups by injected with 3 × 10^−6^ of LM8‐OE‐NC, LM8‐OE‐HTR2B, LM8‐sh‐NC, LM8‐sh‐STAT1‐1, LM8‐sh‐STAT1‐2, LM8‐OE‐NC+sh‐NC, LM8‐OE‐NC+sh‐STAT1, LM8‐OE‐HTR2B+sh‐NC, LM8‐OE‐HTR2B+sh‐STAT1 cells into the left subscapular region. In the BW‐723C86 treatment group, mice were divided into two groups and injected with 3 × 10^−6^ of LM8 cells. When tumors reached 200 mm^3^, one group received intraperitoneal injections of DMSO, and the other group received BW‐723C86 every 2 days for a total of 20 days. Additionally, different doses of BW‐723C86 (10 and 20 mg kg^−1^) were used to evaluate the dose‐response relationships of HTR2B activation in vivo experiment. The survival curve was analyzed according to the survival of mice and the time to reach maximum tumor size. In the RS‐127445 treatment group, mice were divided into two groups and injected with 3 × 10^−6^ of LM8 cells. When tumors reached 200 mm^3^, one group received intraperitoneal injections of DMSO, and the other group received RS‐127445 every 2 days for a total of 20 days. The tumor weight and volume were measured when mice euthanized. Hematoxylin and eosin (HE), Ki67, HTR2B, or STAT1 staining were performed on tumor tissues.

### Single‐Cell Transcriptome Sequencing Analysis

Tumor tissues from C3H mice were processed according to standard protocols: minced, washed, and dissociated into single cells using MojoSort Mouse CD45 Nanobeads (BioLegend, USA). Single‐cell suspensions were analyzed using the 10K Genomics‐PerseusTM according to the manufacturer's instructions. Libraries were sequenced on an Illumina NovaSeq 6000 sequencing system by TENK GENMICS Co. Ltd. (Beijing, China). The 10K Genomics‐Perseus CellCosmo software (https://github.com/10KGenomics/CellCOSMO) for original data filtering, alignment, quantitative, identification of recovery of cells. Finally, gene expression matrix files of individual cells were obtained by transcriptome standardization. Gene expression matrices were generated and analyzed using R software (version 4.1.1) for cell filtration, subpopulation classification, marker gene identification, cell type identification, differential gene analysis, enrichment analysis, pseudotime analysis, and cell communication analysis.

### Statistical Analysis

Statistical analysis was performed using GraphPad Prism 8.3.0 (USA). Data are presented as mean ± SD. Comparisons between two groups were conducted using an unpaired *t*‐test, and the comparisons between multiple groups were conducted using one‐way ANOVA with post hoc Bonferroni correction. A *P*‐value of less than 0.05 was considered statistically significant.

## Conflict of Interest

The authors declare no conflict of interest.

## Author Contributions

Conceptualization: K.Z. and C.Z. performed conception of the hypothesis; Z.H., K.Z., and C.Z. performed study supervision; Z.H., J.Z., J.H., and X.W. performed development of methodology; Z.H., J.Z., J.H. and X.W. performed acquisition of data; Z.H., J.Z., J.H., X.W., K.Z. and C.Z. performed analysis and interpretation of data; Z.H., J.Z., J.H., X.W., K.Z., and C.Z. wrote, review, and/or revision of the manuscript. All authors discuss the study. The manuscript was written through contributions of all authors. All authors have given approval to the final version of the manuscript.

## Supporting information



Supporting Information

## Data Availability

The data that support the findings of this study are available from the corresponding author upon reasonable request.

## References

[1] W. Liu , L. Li , X. Bai , M. Zhang , W. Lv , Y. Ma , Y. Sun , H. Zhang , Q. Jiang , Q. Yao , Z. Y. Zhang , Adv. Sci. 2025, 2409870. 10.1002/advs.202409870 PMC12061288 40056029

[2] R. Ji , Y. Wang , D. Pan , J. Han , Y. Wang , S. Zheng , W. Zhao , X. Li , C. Han , L. Zhang , Cancer Lett. 2024, 591, 216893. 38636892 10.1016/j.canlet.2024.216893

[3] Y. Wang , J. Hong , S. Ge , T. Wang , Z. Mei , M. He , Y. Liu , J. Fang , C. Liu , L. Yang , Y. Yuan , Phytomedicine 2024, 132, 155430. 39047413 10.1016/j.phymed.2024.155430

[4] R. Tatsuno , J. Ichikawa , Y. Komohara , C. Pan , T. Kawasaki , A. Enomoto , K. Aoki , K. Hayakawa , S. Iwata , T. Jubashi , H. Haro , Cell Death Dis. 2024, 15, 108. 38302407 10.1038/s41419-024-06487-y PMC10834992

[5] I. Lilienthal , N. Herold , Int. J. Mol. Sci. 2020, 21, 6885. 32961800 10.3390/ijms21186885 PMC7555161

[6] Z. Jamali , M. Taheri‐Anganeh , Z. Shabaninejad , A. Keshavarzi , H. Taghizadeh , Z. S. Razavi , R. Mottaghi , M. Abolhassan , A. Movahedpour , H. Mirzaei , IUBMB Life 2020, 72, 1306. 32233112 10.1002/iub.2277

[7] Q. Shi , J. Xu , C. Chen , X. Hu , B. Wang , F. Zeng , T. Ren , Y. Huang , W. Guo , X. Tang , T. Ji , Cancer Lett. 2024, 591, 216902. 38641310 10.1016/j.canlet.2024.216902

[8] J. Du , X. Meng , M. Yang , G. Chen , J. Li , Z. Zhu , X. Wu , W. Hu , M. Tian , T. Li , S. Ren , P. Zhao , Adv. Sci. 2025, 12, 2410918. 10.1002/advs.202410918 PMC11948032 39889249

[9] D. K. Tosh , A. Ciancetta , E. Warnick , S. Crane , Z. G. Gao , K. A. Jacobson , J. Med. Chem. 2016, 59, 11006. 27933810 10.1021/acs.jmedchem.6b01183 PMC5201133

[10] L. Peverini , S. Shi , K. Medjebeur , P. J. Corringer , Elife 2024, 12, RP93174. 38913422 10.7554/eLife.93174 PMC11196107

[11] S. Karmakar , G. Lal , Theranostics 2021, 11, 5296. 33859748 10.7150/thno.55986 PMC8039959

[12] L. Chen , S. Huang , X. Wu , W. He , M. Song , Clin. Transl. Med. 2024, 14, 1750. 10.1002/ctm2.1750 PMC11213692 38943041

[13] S. Lin , L. Wang , C. Han , Y. Dai , C. Li , Y. Liu , B. Zhang , N. Huang , A. Zhang , T. Zhang , Y. Wang , J. Xie , H. Tang , Y. Cheng , H. Yao , M. Lou , L. Xue , Z. B. Wu , Neuro. Oncol. 2024, 26, 2010. 38989697 10.1093/neuonc/noae130 PMC11534325

[14] L. Mao , F. Xin , J. Ren , S. Xu , H. Huang , X. Zha , X. Wen , G. Gu , G. Yang , Y. Cheng , C. Zhang , W. Wang , X. Liu , Theranostics 2022, 12, 3928. 35664068 10.7150/thno.70762 PMC9131283

[15] G. Ahangari , H. Norioun , Neuroscience 2025, 569, 184. 39675693 10.1016/j.neuroscience.2024.12.014

[16] J. He , J. Wang , D. Wang , S. Dai , T. Yv , P. Chen , R. Ma , C. Diao , G. Lv , Endocrine 2014, 45, 325. 24078408 10.1007/s12020-013-0050-8

[17] Y. X. Ge , T. W. Zhang , L. Zhou , W. Ding , H. F. Liang , Z. C. Hu , Q. Chen , J. Dong , F. F. Xue , X. F. Yin , L. B. Jiang , Biomaterials 2022, 282, 121407. 35217343 10.1016/j.biomaterials.2022.121407

[18] J. D. Christie , N. Appel , H. Canter , J. G. Achi , N. M. Elliott , A. L. de Matos , L. Franco , J. Kilbourne , K. Lowe , M. M. Rahman , N. Y. Villa , J. Carmen , E. Luna , J. Blattman , G. McFadden , Mol. Ther. Oncolytics 2021, 22, 539. 34553039 10.1016/j.omto.2021.07.014 PMC8433070

[19] Y. Guan , R. Zhang , Z. Peng , D. Dong , G. Wei , Y. Wang , J. Bone Oncol. 2017, 9, 59. 29226090 10.1016/j.jbo.2017.10.002 PMC5715437

[20] D. Zhan , X. Wang , Y. Zheng , S. Wang , B. Yang , B. Pan , N. Wang , Z. Wang , Front. Oncol. 2023, 13, 1147189. 37795441 10.3389/fonc.2023.1147189 PMC10546427

[21] Y. Li , Y. Wang , R. Wu , P. Li , Z. Cheng , Sci. Rep. 2024, 14, 13206. 38851806 10.1038/s41598-024-63896-x PMC11162446

[22] Y. Uchihara , K. Umeda , Y. Yamada , H. Ito , K. Tasaka , K. Isobe , R. Akazawa , N. Kawabata , S. Saida , I. Kato , H. Hiramatsu , T. Noguchi , A. Sakamoto , Y. Arakawa , A. Arakawa , N. Yamamoto , Y. Hosoya , S. Uemura , K. I. Watanabe , H. Sano , T. Taga , J. Takita , Cancer Sci. 2024, 115, 3394. 39080996 10.1111/cas.16297 PMC11447881

[23] W. Xu , Z. Wang , T. Liu , X. Ma , M. Jiao , W. Zhao , L. Yu , Y. Hua , Z. Cai , J. Li , T. Zhang , J. Ethnopharmacol. 2024, 335, 118709. 39163893 10.1016/j.jep.2024.118709

[24] B. K. Nirala , T. Yamamichi , D. I. Petrescu , T. N. Shafin , J. T. Yustein , Cancers 2023, 16, 15. 37894474 10.3390/cancers15205108 PMC10605493

[25] Z. Liao , M. Li , G. Wen , K. Wang , D. Yao , E. Chen , Y. Liang , T. Xing , K. Su , C. Liang , Z. Che , Q. Ning , J. Tang , W. Yan , Y. Li , L. Huang , NPJ Precis. Oncol. 2023, 7, 62. 37386055 10.1038/s41698-023-00415-7 PMC10310742

[26] S. Liu , M. He , H. Sun , Y. Wu , W. Jin , Immune Regul. Therap. Response, Genes 2024, 15, 1541. 10.3390/genes15121541 PMC11675146 39766808

[27] H. Liu , Q. Huang , Y. Fan , B. Li , X. Liu , C. Hu , Acta Pharm. Sin. B 2023, 13, 3400. 37655314 10.1016/j.apsb.2023.05.015 PMC10465950

[28] M. R. Leonard , D. M. Jones , K. A. Read , S. Pokhrel , J. A. Tuazon , R. T. Warren , J. S. Yount , K. J. Oestreich , JCI Insight 2024, 10, 180287. 10.1172/jci.insight.180287 PMC11721307 39560988

[29] H. Ma , T. Wang , J. Wang , P. Wang , Q. Shu , R. Qin , S. Li , H. Xu , Ecotoxicol. Environ. Saf. 2024, 280, 116534. 38823345 10.1016/j.ecoenv.2024.116534

[30] H. He , X. Zhang , H. He , C. Xiao , G. Xu , L. Li , Y. E. Liu , C. Yang , T. Zhou , Z. You , J. Zhang , Int. Immunopharmacol. 2024, 134, 112191. 38759369 10.1016/j.intimp.2024.112191

[31] J. P. Twohig , A. Cardus Figueras , R. Andrews , F. Wiede , B. C. Cossins , A. Derrac Soria , M. J. Lewis , M. J. Townsend , D. Millrine , J. Li , D. G. Hill , J. Uceda Fernandez , X. Liu , B. Szomolay , C. J. Pepper , P. R. Taylor , C. Pitzalis , T. Tiganis , N. M. Williams , G. W. Jones , S. A. Jones , Nat. Immunol. 2019, 20, 458. 30890796 10.1038/s41590-019-0350-0 PMC7610646

[32] C. L. Yang , F. X. Wang , J. H. Luo , S. J. Rong , W. Y. Lu , Q. J. Chen , J. Xiao , T. Wang , D. N. Song , J. Liu , Q. Mo , S. Li , Y. Chen , Y. N. Wang , Y. J. Liu , T. Yan , W. K. Gu , S. Zhang , F. Xiong , Q. L. Yu , Z. Y. Zhang , P. Yang , S. W. Liu , D. Eizirik , L. L. Dong , F. Sun , C. Y. Wang , Mol. Ther. 2024, 32, 2778. 38822524 10.1016/j.ymthe.2024.05.038 PMC11405166

[33] A. Goder , T. Ginter , T. Heinzel , S. Stroh , J. Fahrer , A. Henke , O. H. Kramer , Cytokine 2021, 144, 155552. 34000478 10.1016/j.cyto.2021.155552

[34] A. R. Harrison , K. G. Lieu , F. Larrous , N. Ito , H. Bourhy , G. W. Moseley , PLoS Pathog. 2020, 16, 1008767. 10.1371/journal.ppat.1008767 PMC7480851 32903273

[35] W. Wang , M. C. Lopez McDonald , C. Kim , M. Ma , Z. T. Pan , C. Kaufmann , D. A. Frank , Front. Immunol. 2023, 14, 1265818. 38022653 10.3389/fimmu.2023.1265818 PMC10663227

[36] P. Sun , Z. Li , Z. Yan , Z. Wang , P. Zheng , M. Wang , X. Chang , Z. Liu , J. Zhang , H. Wu , W. Shao , D. Xue , J. Yu , BMC Cancer 2024, 24, 922. 39080642 10.1186/s12885-024-12680-1 PMC11289911

[37] S. K. Yu , T. Yu , Y. M. Wang , A. Sun , J. Liu , K. H. Lu , J. Transl. Med. 2024, 22, 460. 38750462 10.1186/s12967-024-05284-7 PMC11094951

[38] N. N. Khodarev , M. Beckett , E. Labay , T. Darga , B. Roizman , R. R. Weichselbaum , Proc. Natl. Acad. Sci. USA 2004, 101, 1714. 14755057 10.1073/pnas.0308102100 PMC341831

[39] N. Khodarev , R. Ahmad , H. Rajabi , S. Pitroda , T. Kufe , C. McClary , M. D. Joshi , D. MacDermed , R. Weichselbaum , D. Kufe , Oncogene 2010, 29, 920. 19915608 10.1038/onc.2009.391 PMC2820589

[40] S. Wang , C. Darini , L. Desaubry , A. E. Koromilas , Mol. Cancer Ther. 2016, 15, 3055. 27913706 10.1158/1535-7163.MCT-16-0416

[41] P. H. Chou , C. K. Luo , N. Wali , W. Y. Lin , S. K. Ng , C. H. Wang , M. Zhao , S. W. Lin , P. M. Yang , P. J. Liu , J. J. Shie , T. T. Wei , J. Biomed. Sci. 2022, 29, 20. 35313878 10.1186/s12929-022-00803-4 PMC8939146

[42] L. Zhao , X. Li , J. Su , F. W. Gong , J. Lu , Y. Wei , Cell Biochem. Funct. 2020, 38, 630. 32390230 10.1002/cbf.3518

[43] Y. Cheng , P. Wang , L. Liu , J. Environ. Pathol. Toxicol. Oncol. 2024, 43, 53. 39016141 10.1615/JEnvironPatholToxicolOncol.2024052948

[44] B. Dai , M. Fan , X. Huang , Z. Gong , H. Cao , Y. Hu , Q. Su , T. Yang , Y. Chen , X. Peng , F. Liu , Y. Zhang , Phytomedicine 2022, 103, 154249. 35716538 10.1016/j.phymed.2022.154249

[45] W. Ji , Y. Jin , W. Jiang , Crit. Rev. Eukaryot. Gene Exp. 2024, 34, 35. 10.1615/CritRevEukaryotGeneExpr.2024053577 39180206

[46] Y. Zhang , N. Shen , A. Jiang , J. Zhao , Y. Sang , A. Wang , W. Shen , Y. Gao , J. Biomol. Struct. Dyn. 2024, 11, 1. 10.1080/07391102.2024.2318656 38468495

[47] F. Abedini , O. Amjadi , A. Hedayatizadeh‐Omran , S. A. Lira , G. Ahangari , Oncology 2023, 101, 415. 37231904 10.1159/000530878

[48] J. Feng , J. Wang , Y. Wang , X. Huang , T. Shao , X. Deng , Y. Cao , M. Zhou , C. Zhao , Front. Cell Dev. Biol. 2022, 10, 898657. 35874833 10.3389/fcell.2022.898657 PMC9304626

[49] Y. Ping , J. Shan , H. Qin , F. Li , J. Qu , R. Guo , D. Han , W. Jing , Y. Liu , J. Liu , Z. Liu , J. Li , D. Yue , F. Wang , L. Wang , B. Zhang , B. Huang , Y. Zhang , Immunity 2024, 57, 2122. 39208806 10.1016/j.immuni.2024.08.003

[50] T. Feng , Z. Tang , J. Shu , X. Wu , H. Jiang , Z. Chen , Y. Chen , L. Ji , H. Chao , Angew. Chem., Int. Ed. Eng. 2024, 63, 202405679. 10.1002/anie.202405679 38771671

[51] Q. Fan , Y. Wang , J. Cheng , B. Pan , X. Zang , R. Liu , Y. Deng , Front. Immunol. 2024, 15, 1362970. 38629071 10.3389/fimmu.2024.1362970 PMC11018946

[52] F. Li , H. Tang , X. Luo , X. Li , K. Luo , S. Liu , J. Liang , S. Liao , C. Zhong , X. Zhan , Q. Wei , W. Feng , Y. Liu , Cancer Sci. 2023, 114, 3014. 37150900 10.1111/cas.15821 PMC10323104

[53] D. C. Allred , J. M. Harvey , M. Berardo , G. M. Clark , Mod. Pathol. 1998, 11, 155. 9504686

