# Supplementary material for: Activation of HTR2B Suppresses Osteosarcoma Progression through the STAT1‐NLRP3 Inflammasome Pathway and Promotes OASL1+ Macrophage Production to Enhance Antitumor Immunity
Source: Adv Sci (Weinh). 2025 May 19;12(29):e15276. doi: 10.1002/advs.202415276 (PMC12362726; doi:10.1002/advs.202415276)
Supplement: Supplementary file 1 — Supporting Information [file ADVS-12-e15276-s001.docx]

**Supplementary Materials**

Figure S1

**
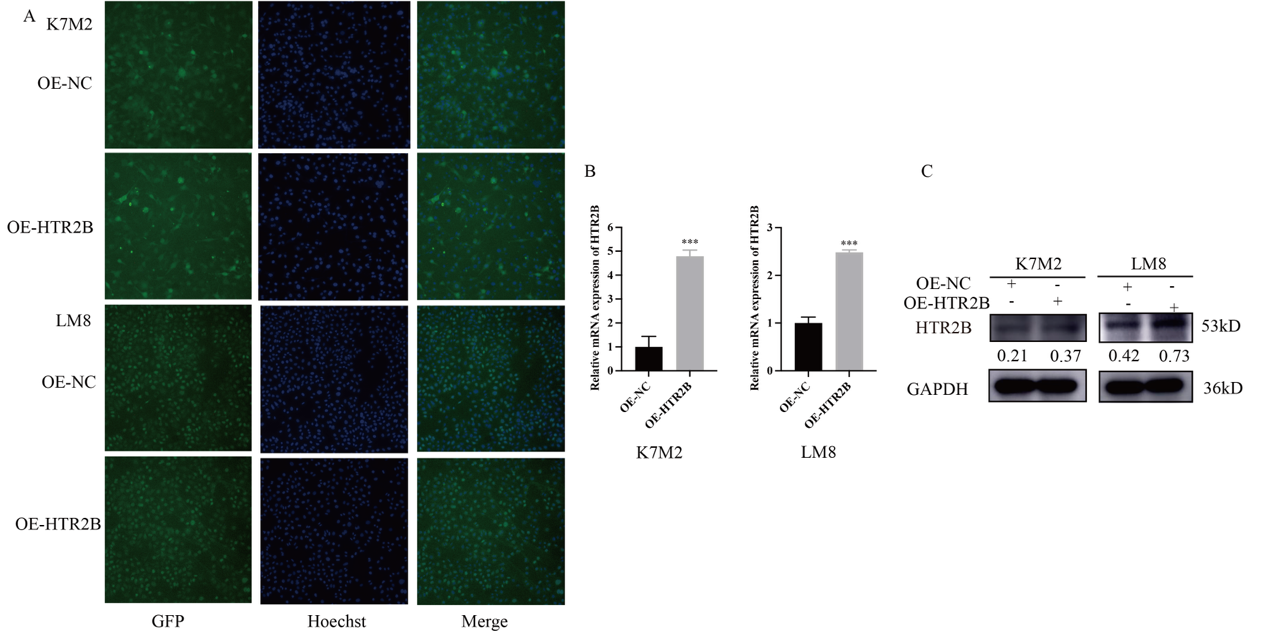
**

Figure S1: HTR2B overexpression lentivirus transfection. (A) Representative images of the OE-NC control group and the OE-HTR2B group after lentivirus transfection of K7M2 and LM8 cells; (B) The RT-qPCR analysis confirmed HTR2B mRNA expression was significantly increased in the OE-HTR2B group; (C) The western blot analysis showed that HTR2B protein was significantly increased in the OE-HTR2B group. ***P<0.001

Figure S2


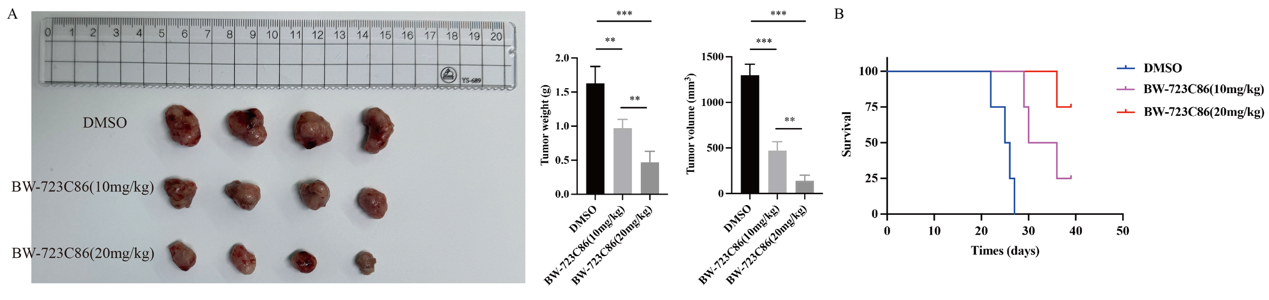


Figure S2: The dose-response relationships of BW-723C86 in vivo experiment. (A) The increased of BW-723C86 dose gradually inhibited tumor growth in vivo; (B) The survival analysis showed that increased of BW-723C86 dose gradually prolonged the survival rates. **P<0.01, ***P<0.001

Figure S3


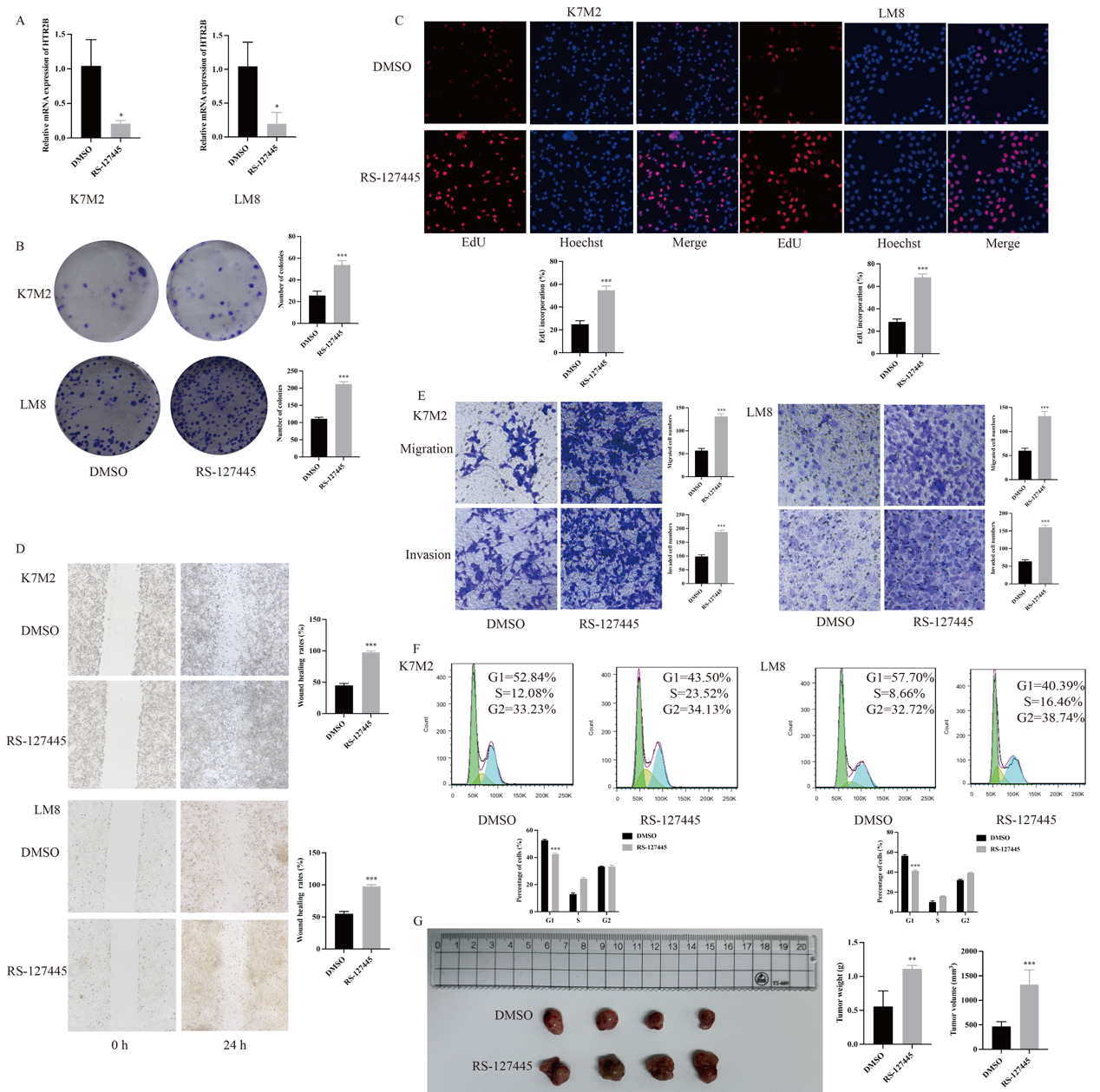


Figure S3: Effects of HTR2B inhibitor (RS-127445) on osteosarcoma proliferation, migration, invasion, cell cycle changes in vitro, and tumor growth in vivo. (A) The RT-qPCR analysis confirmed that HTR2B expression was decreased in RS-127445 treatment group; (B) Colony formation ability of K7M2 and LM8 cells was promoted with RS-127445 treatment; (C) EdU assay revealed that the proliferation capacity of K7M2 and LM8 cells in RS-127445 treatment group was significantly increased; (D) Wound healing assay showed that RS-127445 treatment promoted cell migration in K7M2 and LM8 cells; (E) Transwell assay found that RS-127445 treatment enhanced migration and invasion abilities in K7M2 and LM8 cells; (F) The flow cytometry assay observed reduce proportion of G1 phase in the K7M2 and LM8 cells treated with RS-127445; (G) Tumor growth was significantly promoted in RS-127445 treatment group in vivo. *P<0.05, **P<0.01, ***P<0.001

Figure S4


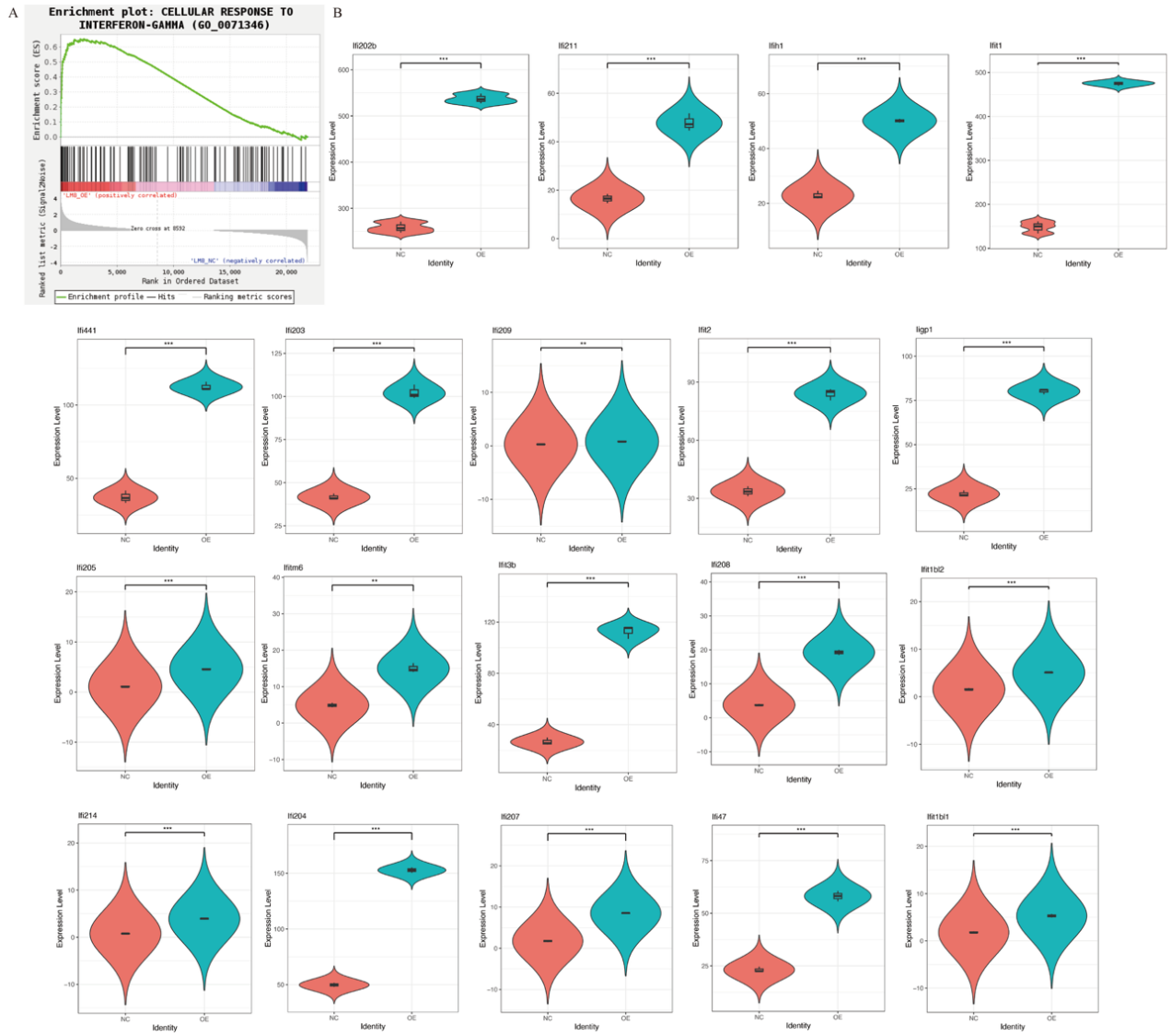


Figure S4: The potential signaling pathway of HTR2B activation promoted STAT1 expression. (A) The GSEA enrichment analysis of transcriptome sequencing found that cellular response to the interferon-gama signaling pathway was enriched; (B) The activation of HTR2B promoted the expression of interferon related genes. **P<0.01, ***P<0.001

Figure S5


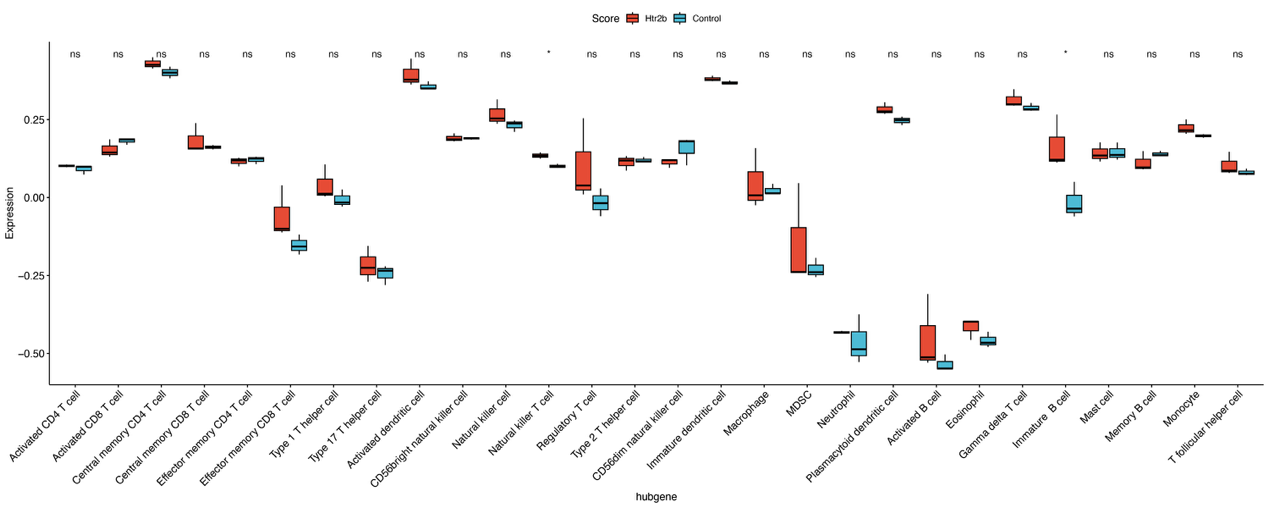


Figure S5: Overexpression of HTR2B promotes immune cell infiltration in osteosarcoma tumor tissues. *P<0.05

Figure S6


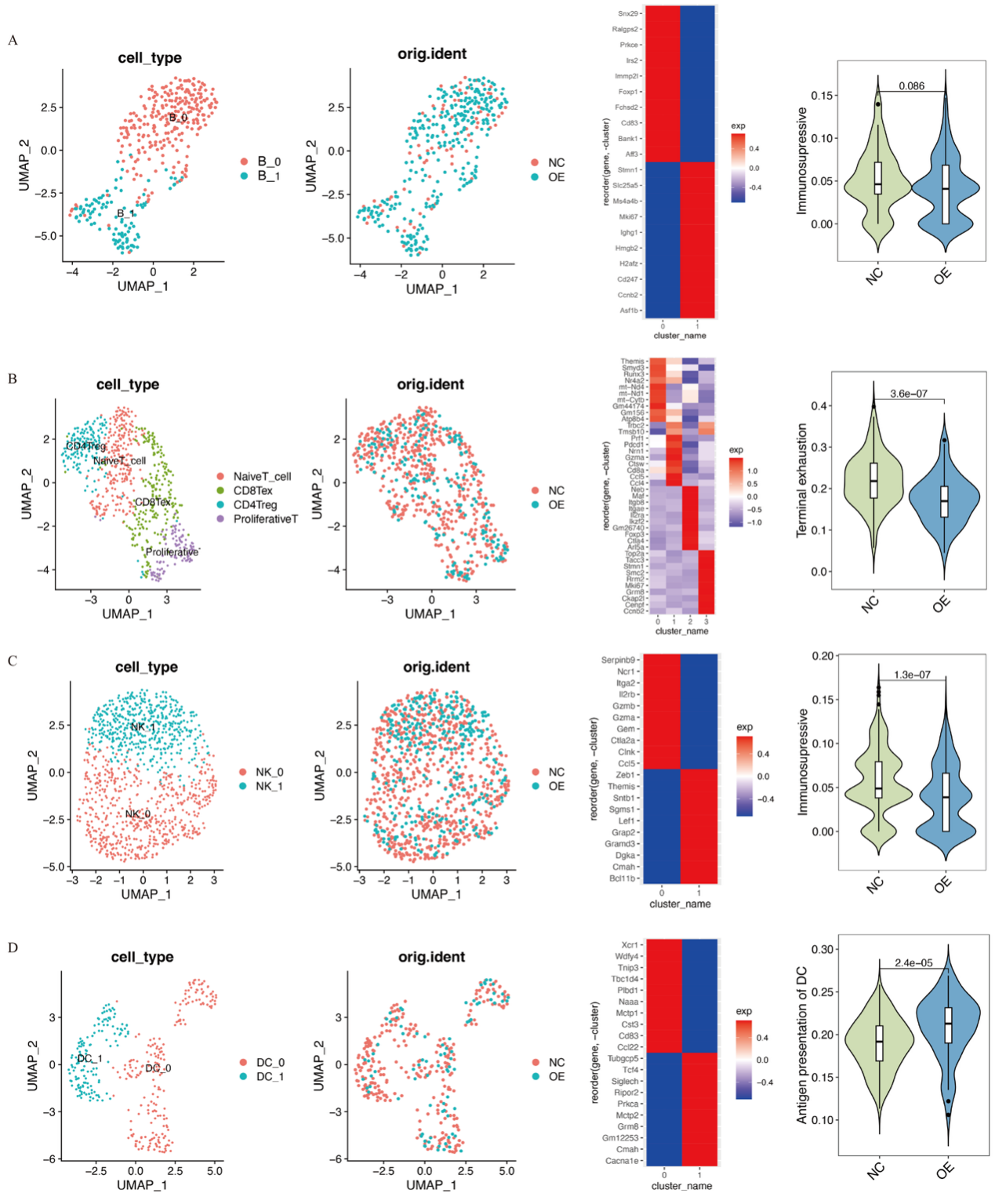


Figure S6: The classification diagrams of T cells, B cells, NK cells, Macrophages, and DC. (A) The B cells subgroup has been defined as B_0 and B_1 clusters, and the distribution of each subgroup in NC and OE groups, the heatmap of markers for B cells subgroups, and the immunosuppressive score was decreased in OE group; (B) The T cells subgroup has been defined as Naive T_cell, CD8 Tex, CD4 Treg and Proliferation T clusters, and the distribution of each subgroup in NC and OE groups, the heatmap of markers for T cells subgroups, and terminal exhaustion score was decreased in OE group; (C) The NK cells subgroup has been defined as NK_0 and NK_1 clusters, and the distribution of each subgroup in NC and OE groups, the heatmap of markers for NK cells subgroups, and immunosuppressive score was decreased in OE group; (D) The DC subgroup has been defined as DC_0 and DC_1 clusters, and the distribution of each subgroup in NC and OE groups, the heatmap of markers for DC subgroups, and antigen presentation of DC score was increased in OE group.

Figure S7


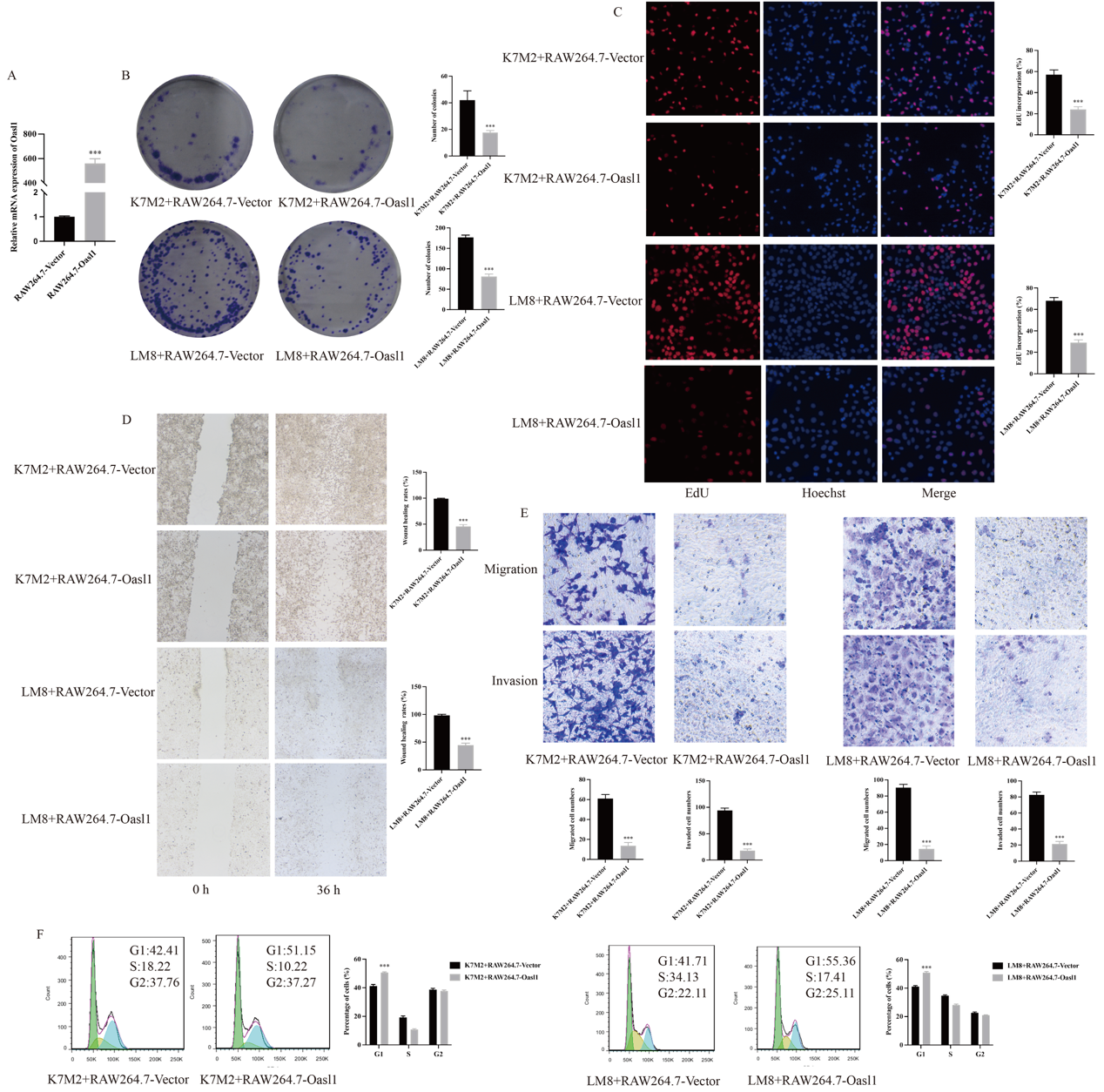


Figure S7: In vitro effects of RAW264.7-Oasl1 on osteosarcoma proliferation, migration, invasion, cell cycle. (A) The RT-qPCR confirmed that Oasl1 expression was significantly promoted in the RAW264.7-Oasl1 group compared to RAW264.7-Vector group; (B) Colony formation ability of K7M2 and LM8 cells was suppressed in co-cultured with RAW264.7-Oasl1 group; (C) EdU assay revealed that the proliferation capacity of K7M2 and LM8 cells was significantly decreased in co-cultured with RAW264.7-Oasl1 group; (D) Wound healing showed that co-cultured with RAW264.7-Oasl1 group suppressed cell migration in K7M2 and LM8 cells; (E) Transwell assay revealed that co-cultured with RAW264.7-Oasl1 reduced migration and invasion abilities in K7M2 and LM8 cells; (F) The flow cytometry assay found that co-cultured with RAW264.7-Oasl1 increased G1 phase proportion in the K7M2 and LM8 cells. ***P<0.001.
